# Transcriptomic and proteomic analyses of core metabolism in *Clostridium termitidis* CT1112 during growth on α-cellulose, xylan, cellobiose and xylose

**DOI:** 10.1186/s12866-016-0711-x

**Published:** 2016-05-23

**Authors:** Riffat I. Munir, Victor Spicer, Oleg V. Krokhin, Dmitry Shamshurin, XiangLi Zhang, Marcel Taillefer, Warren Blunt, Nazim Cicek, Richard Sparling, David B. Levin

**Affiliations:** Department of Biosystems Engineering, University of Manitoba, R3T 5N6 Winnipeg, MB Canada; Department of Physics and Astronomy, University of Manitoba, R3T 5N6 Winnipeg, MB Canada; Manitoba Centre for Proteomics and Systems Biology, University of Manitoba, R3T 5N6 Winnipeg, MB Canada; Department of Plant Science, University of Manitoba, R3T 5N6 Winnipeg, MB Canada; Department of Microbiology, University of Manitoba, R3T 5N6 Winnipeg, MB Canada

**Keywords:** *Clostridium termitidis*, RNAseq, Quantitative proteomics, Biofuel, CAZymes, Metabolism

## Abstract

**Background:**

*Clostridium termitidis* CT1112 is an anaerobic, Gram-positive, mesophilic, spore-forming, cellulolytic bacterium, originally isolated from the gut of a wood feeding termite *Nasusitermes lujae*. It has the ability to hydrolyze both cellulose and hemicellulose, and ferment the degradation products to acetate, formate, ethanol, lactate, H_2_, and CO_2_. It is therefore ges in gene and gene product expression during growth of *C. termitidis* on cellobiose, xylose, xylan, and α–cellulose.

**Results:**

Correlation of transcriptome and proteome data with growth and fermentation profiles identified putative carbon-catabolism pathways in *C. termitidis*. The majority of the proteins associated with central metabolism were detected in high abundance. While major differences were not observed in gene and gene-product expression for enzymes associated with metabolic pathways under the different substrate conditions, xylulokinase and xylose isomerase of the pentose phosphate pathway were found to be highly up-regulated on five carbon sugars compared to hexoses. In addition, genes and gene-products associated with a variety of cellulosome and non-cellulosome associated CAZymes were found to be differentially expressed. Specifically, genes for cellulosomal enzymes and components were highly expressed on α–cellulose, while xylanases and glucosidases were up-regulated on 5 carbon sugars with respect to cellobiose. Chitinase and cellobiophosphorylases were the predominant CAZymes expressed on cellobiose. In addition to growth on xylan, the simultaneous consumption of two important lignocellulose constituents, cellobiose and xylose was also demonstrated.

**Conclusion:**

There are little changes in core-metabolic pathways under the different carbon sources compared. The most significant differences were found to be associated with the CAZymes, as well as specific up regulation of some key components of the pentose phosphate pathway in the presence of xylose and xylan. This study has enhanced our understanding of the physiology and metabolism of *C. termitidis*, and provides a foundation for future studies on metabolic engineering to optimize biofuel production from natural biomass.

**Electronic supplementary material:**

The online version of this article (doi:10.1186/s12866-016-0711-x) contains supplementary material, which is available to authorized users.

## Background

Increases in energy demand, high oil prices, and concerns over climate change are the main driving forces behind renewable alternative fuels [1]. Microbial conversion of sugars, derived from biomass, into biofuels through consolidated bioprocessing (CBP) is a potential alternative to fossil fuels [2]. In CBP, enzyme production, biomass hydrolysis, and biofuel production are all carried out in a single step. However, biomass recalcitrance and low biofuel yields are major challenges that need to be overcome for efficient conversion of biomass derived sugars.

While the genome of an organism can suggest metabolic potential and putative pathways associated with metabolism, high throughput “omics technologies” such as transcriptomics and proteomics [3] provide insights into the selection of genes used for metabolism under specific growth conditions and have been used to understand the genetic and the central carbon metabolism mechanisms for the production of desired end-products in various cellulolytic clostridia cultured on different substrates [4–8]. In addition, studies have previously suggested that *C. thermocellum* modulates cellulosomal subunit composition to better suit the organism’s needs for growth under different conditions [9]. More recently, transcriptome analysis of *C. cellulolyticum* cultured on different substrates [10] revealed substrate specificity of carbohydrate active enzymes (CAZymes) and the transcriptional regulation of core cellulases by Carbon Catabolite Repression (CCR). Under glucose, core cellulases were highly expressed at both transcript and protein levels suggesting that glucose acts as a CCR inhibitor instead of a trigger. Such studies are vital and can be exploited for process and genetic engineering for more effective industrial biofuel production.

*Clostridium termitidis* CT1112 is an anaerobic, Gram-positive, mesophilic, spore-forming, cellulolytic bacterium that has been used previously to study its potential as a candidate for CBP [11, 12]. It can use both simple and complex carbohydrates to carry out mixed-product fermentation, producing acetate, formate, ethanol, H_2_, and CO_2_. The *C. termitidis* genome (GenBank accession number AORV00000000) encodes a variety of carbohydrate-active enzymes (CAZymes), which are spread across various CAZy families. This highlights the potential ability to produce a wide variety of enzymes needed to breakdown different types of complex biomass. In addition, bioinformatic analyses revealed the presence of sequences for cellulosome components (putative dockerin domains, cohesin domains and cellulosome gene clusters), suggesting cellulosome assembly [13]. Cellulosomes have been shown to be highly efficient nano-machines for deconstructing cellulosic substrates [14–17]. We have recently shown that the proteomes of putative cellulosomal enzymes were up regulated in *C. termitidis* cultured on α-cellulose compared to cellobiose [18]. This suggests that, as with other anaerobic cellulolytic bacteria*, C. termitidis* also requires the use of cellulosomes to deconstruct complex polysaccharides such as cellulose [1, 19–22]. No studies however, have been reported so far on the expression of genes involved in *C. termitidis* substrate hydrolysis and metabolism.

In this study, *C. termitidis* was cultured on lignocellulose derived simple and complex carbohydrates: cellobiose, xylose, xylan, and α–cellulose as sole carbon sources. RNA seq transcriptome profiles (next generation sequencing to identify and quantify RNA in biological samples) and 4-plex 2D HPLC-MS/MS quantitative (iTRAQ) proteomic profiles were analyzed to identify the genes involved in substrate degradation, cellodextrin transport and end product synthesis related genes. Identification of these genes is important in understanding the metabolic networks of *C. termitidis* and could be valuable engineering targets for improving biomass to biofuel production.

## Results and discussions

### Growth and end-product synthesis

**Fig. 1:**
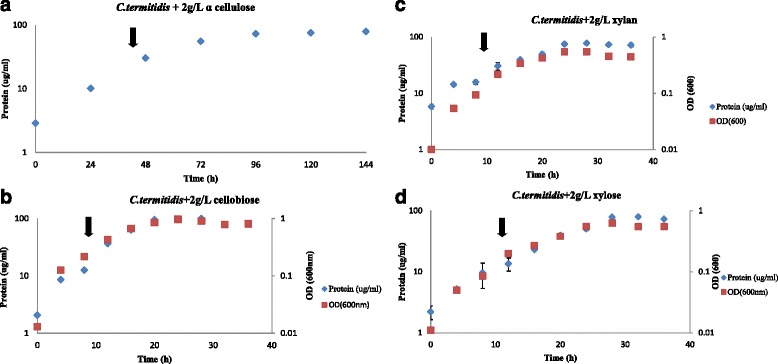
Growth curves for *C. termitidis* cultured on 2 g/L each of α-cellulose (**a**), cellobiose (**b**), xylan (**c**) and xylose (**d**). Arrows indicate the time points sampled for proteomics and transcriptomic analysis. Error bars too small to be visible

Cell growth of *C. termitidis*, based on OD_600_ values, protein concentration, substrate consumption and end-product formation during growth on the four substrates (2 g/L each of cellobiose, xylose, α-cellulose and beechwood xylan), is shown in Fig. 1 and Additional file 1. Because cells used for various inoculations were grown on the same medium with the same substrate, no lag in growth was observed under all conditions tested. *C. termitidis* remained in exponential phase for up to approximately 80 h on α-cellulose. On both xylan and cellobiose the cells were in exponential phase for up to approximately 20 h, and up to 24 h on xylose. The generation times, calculated based on growth in exponential phase, were 19.5 h per generation for α-cellulose, 4.7 per generation for cellobiose, and 4.8 h per generation for xylose. In the case of xylan, a generation time of 5.5 h was observed, a value which is more comparable to those reported for soluble substrates.

Hydrogen, CO_2_, lactate, formate, acetate and ethanol were identified as the major fermentation end-products and their synthesis patterns correlated with cell growth under all conditions (Additional file 1). In cultures containing cellobiose or xylose, acetate, and formate were the major soluble end products. Acetate, formate and lactate production increased sharply with a drop in pH. However, in cultures containing insoluble substrates (α-cellulose and xylan), acetate was the dominant soluble end-product during late exponential phase and stationary phase of growth. Overall average carbon recovery for xylose and cellobiose was 0.87 and 0.89 respectively. Oxidation:reduction ratios for α-cellulose, cellobiose, and xylose were calculated to be 0.96, 0.93, and 0.87, respectively, indicating that most of the major end-products were accounted for. The carbon balance for xylan cultures could not be completed due to lack of adequate quantification methodology. Samples for proteomics and RNA sequencing were taken at exponential phase of growth as indicated by the arrows in Fig. 1.

### RNA sequencing and proteomics (Overall findings)

RNA sequencing and iTRAQ 2D-HPLC MS/MS were carried-out with *C. termitidis* cultures under 4 different substrate conditions, to identify changes in gene and protein (gene product) expression profiles, respectively. For RNA, linear regression analysis of log2 of the sum of 100-mer alignment elements shows good correlation (*R*^2^ > 0.9) between biological replicates under all conditions tested (Additional file 2). This suggests a high degree of reproducibility between the two independently replicated experiments with respect to stability, sample preparation, sequencing, and analysis. Furthermore, scatter plot analysis of intra-replicate and across state (between substrates) RNA Z-scores indicated differences in gene expression when moving from one state to another. However, very little variation was observed between biological replicates (Additional file 3).

Overall, of the 5389 total genes encoded by the *C. termitidis* genome, differential RNA seq analysis successfully aligned approximately 95 % of the genes in the various substrates, indicating that RNA seq analysis achieved a comprehensive coverage of the *C. termitidis* transcriptome.

For proteomic data analysis, 278,048 MS/MS spectra identified 95,934 peptides (22,518 non-redundant) spanning 2345 proteins with expectation values log(e) < −10 (a 1-in-10-billion probability that the proteins identified have a better random answer). Even though RNA seq and proteomics offered different degrees of gene access, overall analysis showed a similar trend in gene and gene product expression (TIC and RNAseq expression values for a particular gene). The log2 expression values of RNA seq and proteomic data largely correlated with each other, with R^2^ values ranging between 0.3 and 0.37 (Additional file 4). This suggests that while the full depth of the differential analysis was best achieved with the transcriptomic data, the proteomic data could be used to supporting/ confirming the transcriptomic data, provided the proteins detected were sufficiently abundant. While data obtained from both technologies is presented, RNA sequencing data was mainly used for analysis with the proteomics data only used for confirmation/supporting purposes. The overall biological signal to systematic noise ratio of RNAseq analyses under four experimental conditions is presented in Additional file 5. Differences observed were more pronounced between α–cellulose vs cellobiose, followed by xylan vs cellobiose. Xylose and cellobiose showed the least differential expression variation. The RNA seq expression values, TIC values, Znet scores, and S/N ratios on a single gene level identified in *C. termitidis* cultured under various substrate conditions is provided in Additional file 6.

An overview of the relative changes in expression, observed in the outermost 10 % of the populations (Z-scores magnitudes ≥ 1.65) is given in Table 1. Genes and proteins were grouped according to their respective COG designation to identify responses to changing substrate conditions. The most pronounced changes, in terms of number of genes/proteins affected, were observed in COG Class G (Carbohydrate Metabolism and Transport). Members in this category are predominantly involved in the degradation and metabolism of lignocellulosic substrates, and thus there were more up-regulated members than down-regulated in the substrates tested compared to cellobiose. Other categories that were also well represented, with 10 or more members under specific substrate conditions, include COG classes M (Cell wall/membrane/envelope biogenesis), E (amino acid transport and metabolism), Q (Secondary metabolites biosynthesis, transport and catabolism), R (General function prediction), and S (Function unknown).

**Table 1:** Number of genes and proteins in COG classes up- and down-regulated^a^ on various substrates compared to cellobiose

COG class	Description	Xylose vs cellobiose genes		Xylose vs cellobiose proteins		Xylose vs cellobiose (overlap of genes and proteins)		α-cellulose vs cellobiose genes		α-cellulose vs cellobiose proteins		α-cellulose vs cellobiose (overlap of genes and proteins)		Xylan vs cellobiose genes		Xylan vs cellobiose proteins		Xylan vs cellobiose (overlap of genes and proteins)	
		Down	Up	Down	Up	Down	Up	Down	Up	Down	Up	Down	Up	Down	Up	Down	Up	Down	Up
C	Energy production and conversion	3	1				1	2	1	3	1	2	2	3	1	2	0	1	1
D	Cell cycle control, cell division, chromosome partitioning	2	0					1	2										
E	Amino acid transport and metabolism	8	14	2	4	1	4	4	8	0	3	1	1	4	0	2	0	2	1
F	Nucleotide transport and metabolism							0	2										
G	Carbohydrate transport and metabolism	11	32	3	23	7	27	5	28	3	20	1	33	7	40	5	24	7	40
H	Coenzyme transport and metabolism	4	3					1	6	0	2		3	2	0			1	3
I	Lipid transport and metabolism	7	1	2	0	1		1	3	1	2	1	1	4	0	4	0	3	
J	Translation, ribosomal structure and biogenesis									4	1	1							
K	Transcription	6	1			1	4	4	6	1	3	1	2	1	2	3	1	1	1
L	Replication, recombination and repair	3	1			1													
M	Cell wall/membrane/envelope biogenesis	3	1					1	15	1	9	1	4	1	2				
N	Cell motility							3	1	2	1	1							
O	Posttranslational modification, protein turnover, chaperones	2	1					3	4	2	5	1	4						
P	Inorganic ion transport and metabolism	2	4	2	1		1	1	4			1	1	2	0				
Q	Secondary metabolites biosynthesis, transport and catabolism	19	1	6	0	5		1	7	2	0	1	1	15	0	7	0	5	2
R	General function prediction only	6	9	3	2	1	3	3	17	1	4	1	5	2	7	3	2	2	6
S	Function unknown	6	0	0	3			1	11	3	4			2	1	3	0		
T	Signal transduction mechanisms	2	5				2	3	4	3	1	1	1	0	3				1
U	Intracellular trafficking, secretion, and vesicular transport	2	0					2	0										
V	Defense mechanisms							2	3	2	0		1						
TOTAL		86	74	18	33	17	42	38	122	28	56	14	59	43	56	29	27	22	55

Overlap of differentially expressed genes and proteins with Z-score magnitudes of ≥ 1.65 were manually assessed and do not include genes in category S and hypotheticals

^a^Up- and down-regulation enumeration uses Z-scores with magnitudes ≥ 1.65, approximately representing outermost 10 % of the population

### Cellulose and hemicellulose degradation and transport

#### Transcription of cellulosomal components

Analysis of the *C. termitidis* CT1112 genome has so far revealed the presence of 33 cellulosomal genes, which were all transcriptionally identified in the current study (Table 2). The cellulosomal enzymes detected have diverse putative endo- and exo-glucanase activities against a variety of biomass components and includes cellulases, xylanases, mannase, polysaccharide lyases, and esterases. Similar to our previous study [18], the overall Znet-score values suggest that the cellulosome associated genes (R1) and proteins (P1) were significantly up-regulated on α–cellulose vs cellobiose as compared to other substrates. This indicates that, as with other cellulosome forming cellulolytic bacteria such as *C. thermocellum*, the cellulosome system of *C. termitidis* is crucial for substrate hydrolysis, and perhaps the main contributor in the degradation of cellulosic substrates by *C. termitidis*. Furthermore, the major cellulosomal cellulases - exoglucanases Cter_0524 (GH 48) and Cter_0521 (GH 9) - were the two most abundantly expressed cellulosomal transcripts identified on α–cellulose. GH48 (CelS) and GH9 (CbhA) have been similarly reported to be among the highly expressed genes in *C. thermocellum* cultured on insoluble cellulosic substrates [4, 5]. Endoglucanases Cter_0519 (GH5), Cter_0523 (GH8), Cter_4545 (GH9), and Cter_0522 (GH9) were also among the top 10 highly abundant carbohydrate active enzyme genes on α–cellulose (RNA expression values >11). All the non-catalytic cohesion domain containing cellulosomal genes (Cter_0001, Cter_3731, Cter_0520, Cter_0525, Cter_0526) were similarly up-regulated on α–cellulose and were among the highly expressed genes on a transcriptional level (Additional file 6).

**Table 2:**
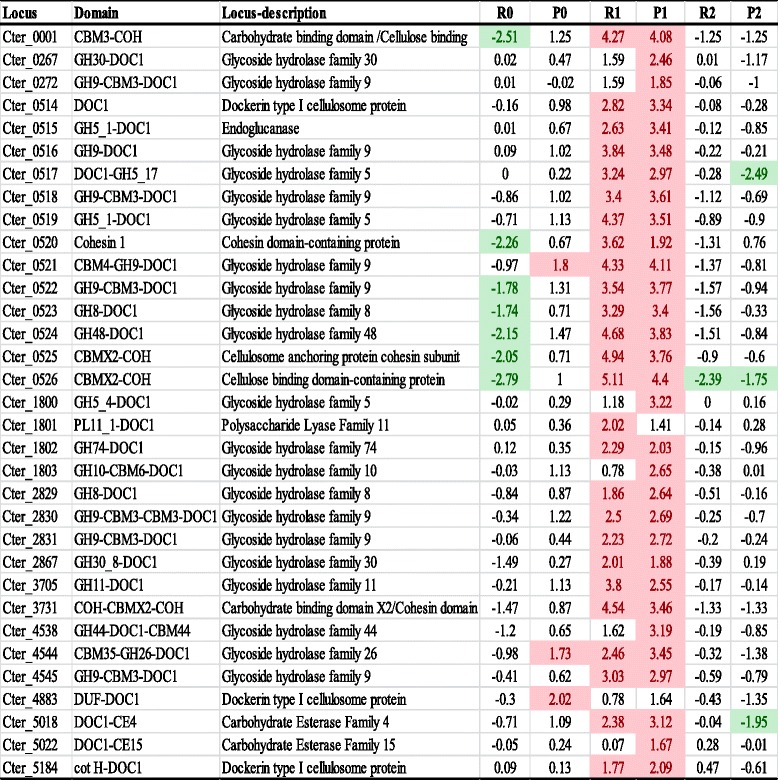
Znet-scores (normalized differential values) of putative cellulosomal genes identified in *C. termitidis* cultured under various substrate conditions and compared to cellobiose

Transcriptomic Z-scores Rnet are represented as: (i) R0: xylose grown cells – cellobiose grown cells; (ii) R1: α-cellulose grown cells – cellobiose grown cells; and (iii) R2: xylan grown cells – cellobiose grown cells. Proteomic Z-scores Pnet are represented as: (i) P0: xylose grown cells – cellobiose grown cells; (ii) P1: α-cellulose grown cells – cellobiose grown cells; and (iii) P2: xylan grown cells – cellobiose grown cells. Z-scores of ≥1.65, up regulated in the corresponding substrate with respect to cellobiose are highlighted in red. Z-scores of ≤ −1.65, down regulated in the corresponding substrate with respect to cellobiose are highlighted in green. For any protein or RNA transcripts, a negative value represents higher expression on cellobiose, while a positive value represents higher expression in any of the other corresponding substrate. For any protein or RNA transcripts, a negative value represents higher expression on cellobiose, while a positive value represents higher expression in any of the other corresponding substrate. DUF: domain of unknown function; Cot H: spore coat protein H; DOC1: dockerin type 1; COH: cohesion domain

Of the 33 cellulosomal genes, four gene clusters were identified which include: (i) an approximately 20 kbp cellulosomal cluster (Cter_0514-Cter_0526), which is similar to the gene cluster that encodes major cellulosome components in other anaerobic, mesophilic, cellulosome-forming cellulolytic Clostridia, such as *C. cellulovorans* and the *cip-cel* cluster in *C. cellulolyticum* [23–25]; (ii) a second cluster of 4 genes (Cter_1800-Cter_1803), which mainly encodes putative hemicellulases (xylanases); (iii) a 3 gene cluster (Cter_2829-Cter_2831) with putative cellulase activities; and (iv) a 2 gene cluster (Cter_4544-Cter_4545) similar to Ccel_0752-Ccel_0753 cluster in *C. cellulolyticum* [10], with putative activities against mannans and cellulose. Interestingly, the hemicellulase cluster Cter_1800-1803, as with some other xylanases indicated below, was up-regulated on α–cellulose compared to xylan. This may putatively indicate the ability of *C. termitidis* to target both five and six carbon sugars simultaneously. The transcriptome Z-scores of xylose versus cellobiose (R0) were reversed in the proteome (P0), which showed up-regulation on xylose relative to cellobiose. However, the genes on these two substrates were expressed at very low levels and therefore do not indicate any significant changes.

#### Transcription of non-cellulosomal carbohydrate active enzymes

In addition to cellulosomal enzymes, the *C. termitidis* genome is known to harbor a variety of non-cellulosomal CAZymes, potentially required for hydrolysis of a variety of carbohydrates. Table 3 shows transcriptional Z-scores of non-cellulosomal CAZymes identified in the outer most 10 % of the population (standard deviation ≥ 1.65) on different substrates compared to cellobiose.

**Table 3:**
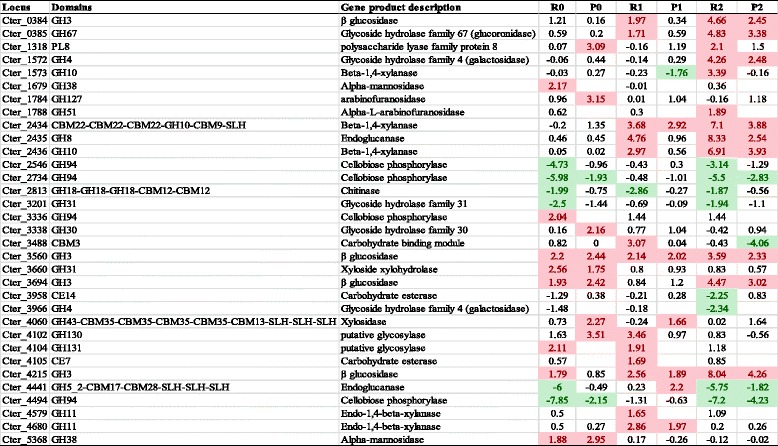
Znet-scores (normalized differential values) of highly expressed non-cellulosomal CAZymes identified in *C.termitidis* cultured under various substrates with respect to cellobiose

Values shown are limited to Z-scores with magnitudes outside ≥ 1.65 (representing outermost 10 % of the population) under any one substrate condition. Transcriptomic Z-scores Rnet are represented as: (i) R0: xylose grown cells – cellobiose grown cells; (ii) R1: α-cellulose grown cells – cellobiose grown cells; and (iii) R2: xylan grown cells – cellobiose grown cells. Proteomic Z-scores Pnet are represented as: (i) P0: xylose grown cells – cellobiose grown cells; (ii) P1: α-cellulose grown cells – cellobiose grown cells; and (iii) P2: xylan grown cells – cellobiose grown cells. Z-scores of ≥1.65, up regulated in the corresponding substrate with respect to cellobiose are highlighted in red. Z-scores of ≤ −1.65, down regulated in the corresponding substrate with respect to cellobiose are highlighted in green. For any protein or RNA transcripts, a negative value represents higher expression on cellobiose, while a positive value represents higher expression in any of the other corresponding substrate

The transcription of some non-cellulosomal CAZyme genes in *C. termitidis* appeared to be substrate-dependent. GH94 (cellobiose phosphorylases Cter_4494, Cter_2734, Cter_2546), GH31 (α-glucosidase Cter_3201), and multi-domain GH18 (Chitinase Cter_2813) were the top 5 highly expressed enzymes in cultures containing cellobiose and were relatively up-regulated on this substrate compared to other substrates. Cellobiose phosphorylases were also found to be the most abundantly expressed CAZymes in cellobiose cultures of *C. thermocellum* [6] and may be involved in intracellular phosporylytic cleavage of cellobiose and other cellodextrins.

Other non-cellulosomal CAZymes up-regulated in cultures containing other substrates compared to cellobiose included polysaccharide lyases, glucosidase, glucoronidase, endoglucanases, arabinofuranosidases, and xylanases. Multi-domain endo-glucanase GH5 (Cter_4441) was found in high abundance and up–regulated on α-cellulose, as were a number of xylanases including the xylanase cluster Cter_2434-Cter_2436, which was also highly expressed and up-regulated on xylan. Genome analysis showed that a gene (Cter_2433) adjacent to the cluster (Additional file 6) encodes for a putative fibronectin type-3 homology domain (Fn3). Fn3 proteins have been implicated in modifying cellulose surface and aid in its hydrolysis by *C. thermocellum* [26]. Because of a similar pattern of expression, it is possible that Cter_2433 forms an operon with the xylanase cluster (Cter_2434-Cter_2436) and is thus expressed highly on both polysaccharides. Nevertheless, expression of hemicellulases under both cellulose and hemicellulose conditions suggests a probable means by which *C. termitidis* prepares for mining energy from natural insoluble substrates.

#### Regulation of carbohydrate active enzymes

Catabolite control protein A (CcpA) has been associated with carbon catabolite repression (CCR) in *Bacillus subtilis* [27, 28]. Analysis of the *C. termitidis* genome identified genes encoding a CcpA-dependent CCR. Four genes encoding putative histidine containing Hpr family of proteins Cter_1040, Cter_3631, Cter_4771 and Cter_4826 were identified. In addition, BLAST analyses revealed the presence of 15 genes (assigned to COG 1609) in *C. termitidis* that were homologous to the CcpA LacI transcriptional regulators of *B.subtilis* (strain 27E1) (Additional file 7).

In order to identify the CAZyme genes that may possibly be regulated by a CCR mechanism, 135 sites homologous to a *B. subtilis cre* consensus sequence were identified (Additional file 8). However, only 6 of these putative *cre* sequences were found adjacent to or overlapping with genes encoding non-cellulosomal CAZymes. A *cre* sequence has been implicated in the regulation of the cellulosomal *cipC* cluster in *C. cellulolyticum* [29]. We were unable to identify any *cre* sequences adjacent to any genes encoding cellulosomal components. In the case of *C. termitidis* cellulosome cluster, this may be due to the fact that the DNA sequence up-stream is truncated and we may have missed a possible *cre* sequence, or it may suggest that cellulosomal genes might be regulated by a CcpA-independent mechanism.

Two-component regulatory systems (TCSs) have been implicated for their role in signal transduction in many prokaryotes. TCSs consist of a membrane bound histidine kinase that senses a specific environmental stimulus, and a response regulator that mediates a cellular response to changes in environmental conditions through expression or repression of target genes [30]. Previous transcription studies on *C. cellulolyticum* have shown that TCS transcriptionally regulate several cellulosomal and non-cellulosomal CAZymes [10, 31]. Analysis of the *C. termitidis* genome revealed the presence of more than a hundred putative TCS regulatory genes. Further analysis showed that many of these might be involved with the regulation of various CAZymes. This hypothesis is supported by the observation that a number of TCSs genes are surrounded by genes encoding both ABC sugar transporters and CAZyme genes, and that these loci exhibit similar patterns of mRNA expression (high or low) under the carbon sources tested, putatively suggesting co-regulation by TCSs (Additional file 6).

The majority of these CAZyme genes encode non-cellulosomal enzymes and include cellulase (GH5: Cter_2349), glucosidase (GH3: Cter_3560), xylanases (GH8: Cter_1787; GH10: Cter_1573, 2434, 2435, 2436), xylosidases (GH35: Cter_0468; GH43: Cter_1576, 2817, 3581, 4113, 4114; GH39: Cter_1786; GH31: Cter_3663, 5208), galactosidases (GH35: Cter_0468, GH2: Cter_4240, GH4: 1572, 1402), mannanases (GH76: Cter_5351; GH 38: 2757) arabinofuranosidases (GH51: Cter_3501, 1788), and cellobiose phosporylase (GH94: Cter_3440). In addition to non-cellulosomal enzymes, 5 genes that encode cellulosomal components were identified, which may putatively be regulated by TCSs. These include the hemicellulase cluster Cter_1800-1803 and the cohesion domain encoding Cter_3731. However no ABC transporters were found associated with these genes, and transport of hydrolysis products may rely on the use of independently transcribed ABC transporters.

#### Transcription of cellodextrin transport related genes

The *C. termitidis* genome encodes for over 700 ABC type transporter components, which are putatively involved in the transport of oligosaccharides, antimicrobials, amino acids, and metal ions. In this study, 8 gene clusters encoding ABC-type systems for transport of sugars were expressed at high levels (among the top 10 % expressed under any one condition tested) (Additional file 9). Gene cluster Cter_4496 - 4498, the products of which were also identified previously [18], was highly up-regulated on cellobiose compared to both xylan and xylose (the 5 carbon sugar substrates). Two genes, Cter_4494 (cellobiose phosphorylase) and Cter_4495 (a transcriptional regulator) adjacent to the cluster were also up-regulated on cellobiose. This may suggest the involvement of the gene cluster for import and subsequent phosphorolytic cleavage of oligosaccharides and cellobiose.

Three ABC transport gene clusters (Cter_2437 – 2439; Cter_3554 – 3557; and Cter_0390 – 0391), which were also detected in our previous proteomic study, were highly expressed and up regulated in cultures containing xylan and α–cellulose, and were up-regulated in cultures containing xylan, α–cellulose, and xylose compared to cellobiose. This may suggest that *C. termitidis* has the probable ability for uptake and utilization of cellodextrins that are longer than cellobiose. Cter_2437 - 2439 lies next to the xylanase cluster Cter_2434 - 2436 and may be responsible for uptake of xylan hydrolysis products. Its expression on α–cellulose correlates with the high expression of the xylanase cluster on the same as indicated above.

Transcripts for four highly expressed ABC transport gene clusters Cter_5427-5429, Cter_0261- 0262, Cter_1947-1949 and Cter_0914 - 0916 were found to be more responsive towards xylose cultures. The gene products of Cter_1947 and Cter_19478 however were not detected and thus may not be involved in sugar transport. Even though Cter_0914 - 0916 encodes a putative ABC type system for xylose transport, it was also expressed highly on α–cellulose compared to cellobiose.

### Ability to utilize 5 and 6 carbon sugars simultaneously

**Fig. 2:**
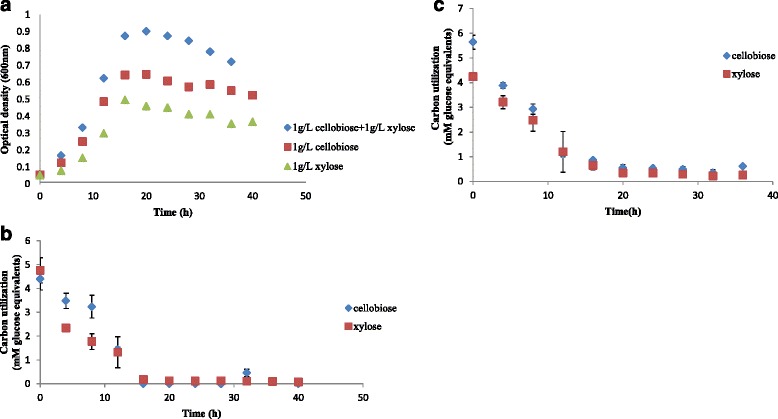
Growth curve (**a**) and substrate consumption of *C. termitidis* cultured on 1 g/L each cellobiose and xylose (**b**), and 1 g/L cellobiose plus 1 g/L xylose (**c**). Reported values are averages of three biological replicates

The expression of genes and gene products for hemicellulases and ABC type systems for xylose transport, in cultures containing α–cellulose suggests that in the natural environment, *C. termitidis* is possibly primed and ready to attack and utilize both cellulose and hemicellulose components concomitantly. This led us to evaluate the substrate utilization profiles of *C. termitidis* cultured on xylose and cellobiose, as single (xylose or cellobiose) compared with mixed (xylose plus cellobiose) carbon sources. The growth curves of *C. termitidis* under these culture conditions are shown in Fig. 2a. Cultures showed no lag phase in growth on all substrates. Xylose and cellobiose cultures grew to an average maximum OD_600_ of 0.50 and 0.66 respectively, while cultures on cellobiose plus xylose grew to a maximum OD_600_ of 0.90. Generation times closely corresponded to each condition, with xylose, cellobiose, and cellobiose plus xylose having an average generation times of 4.8 ± 0.2 h/generation, 4.5 ± 0.25 h/generation, and 4.1 ± 0.33 h/generation, respectively.

As previously seen with 2 g/L substrate concentrations, the major fermentation end-products synthesized during growth included H_2_, CO_2_, lactate, formate, acetate, and ethanol, and are shown in Additional file 10. Since 1 g/L cellobiose plus 1 g/L xylose amounts to 2 g/L substrate, the end-product concentrations corresponded to concentrations seen previously on 2 g/L individual cultures of xylose and cellobiose. As expected, lower end-product concentrations were observed in cultures containing single substrates. Average carbon recovery for cultures on cultures containing xylose, cellobiose, and xylose plus cellobiose were found to be 0.80, 0.98, and 0.80, respectively, and the corresponding OR balances were 0.81, 0.80, and 0.92, respectively. This suggests that the major end-products were accounted for under each condition. Substrate utilization profiles of *C. termitidis* grown on single and mixed substrates (Fig. 2b and c) indicate that both xylose and cellobiose were consumed steadily and simultaneously by *C. termitidis* when cultured in mixed substrates. This is supported by the fact that the enzymes involved in the xylose degradation pathway (discussed below) are not inhibited, as indicated by their transcriptomic and proteomic profiles, when the cells are growing on hexoses alone (both cellobiose and α –cellulose). Similarly growth on xylose and xylan does not inhibit the glycolytic pathways.

### Central metabolism and end product synthesis genes

#### Glycolysis

**Fig. 3:**
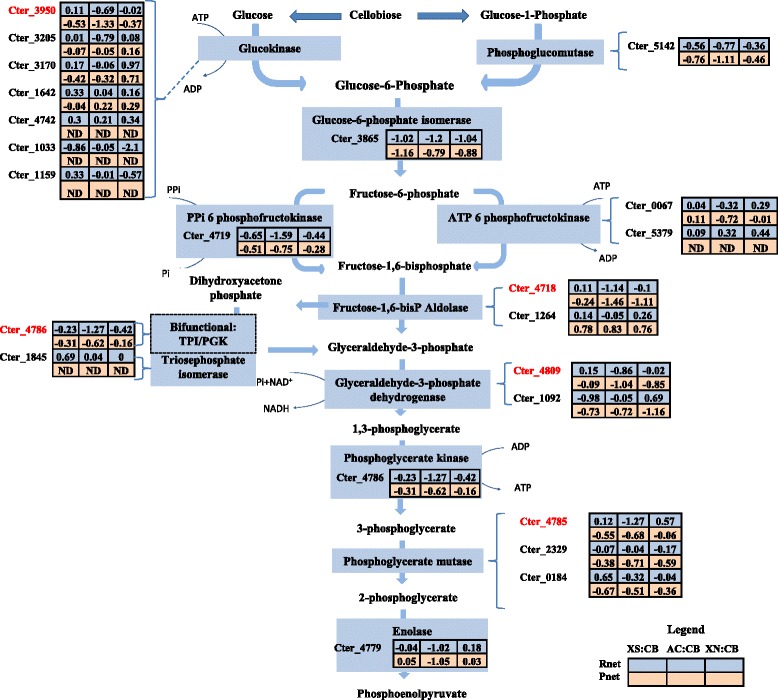
Normalized differential values (Znet-scores) of both the transcriptome (Rnet) and the proteome (Pnet) of glycolysis reactions in *C. termitidis*. TPI: triosephosphate isomerase; PGK: phosphoglycerate kinase; ND: not detected; XS: xylose; CB: cellobiose; AC: α-cellulose; XN: xylan. Locus tags in red indicate high abundance in comparison to paralogs

Analysis of the *C. termitidis* genome suggests that six carbon sugars are converted to phosphoenolpyruvate (PEP) via the Embden-Meyerhof-Parnas (EMP) pathway (shown in Fig. 3). All the glycolytic genes and gene products were detected in high abundance under all the substrate conditions tested, with a few exceptions (Additional file 11). Overall, the Z-scores (Rnet and Pnet) suggest slight up-regulation of these on cellobiose compared to other substrates, a significant difference was however not observed between substrates. Of the seven genes encoding glucokinases, the gene products of only four genes (Cter_3950, Cter_3170, Cter_3205, Cter_1642) were detected with Cter_3950 being the most abundant. The *C. termitidis* genome encodes three putative 6-phosphofructokinases for the inter-conversion of fructose-1-phosphate to fructose-1,6-bisphosphate (EC:2.7.1.11), of which Cter_4719 is putatively pyrophosphate-dependent (PPi) and, Cter_0067 and Cter_5379 are ATP-dependent. Cter_4719 was the most abundant with RNA transcript expression values ranging from 12.5 to 14.25 and the proteomic TIC values of 20.7–21.2, while the transcription of Cter_5379 was near the minimum detection level (RNA expression = 0–1.5) (Additional file 11). Cter_0067 was also expressed abundantly, however in lower quantities than Cter_4719. This may suggest that inter conversion of fructose-1-phosphate to fructose-1,6-bisphosphate can occur by both PPi- and ATP-dependent phosphofrutokinases. Cter_1845, which encodes for triosephosphate isomerase, was detected at very low levels in the transcriptome. However, Cter_4786, which is annotated as a bifunctional triosephosphate isomerse/phosphoglycerate kinase, was expressed abundantly under all conditions in the transcriptome and the proteome and putatively functions both as a triosephosphate isomerase and phosphoglyceratekinase. A similar bifunctional triosephosphate isomerse/phosphoglycerate kinase has been identified and characterized in the hyperthermophilic bacterium *Thermotoga maritima* [32], where it was found that the two enzymes are covalently linked to form a bifunctional fusion protein with dual enzyme activities. Of the two genes encoding fructose-1,6-P aldolases, Cter_4718 was among the top 10 % and was detected at higher levels than Cter_1264, while glyceraldehyde-3-P dehydrogenase (Cter_4809) was the most highly expressed of all the genes involved in carbon metabolism with both the genes and its products detected in the top 10 of all genes expressed. Studies in *C. thermocellum* by Rydzak et al. [6], similarly identified glyceraldehyde-3-P dehydrogenase as the most abundantly expressed protein during growth on cellobiose. Of the multiple copies of phosphoglycerate mutase present in the genome of *C. termitidis*, Cter_4785 showed a much higher level of expression than its counterparts and may be putatively responsible for the conversion to 2-phosphoglycerate.

#### Pentose phosphate pathway

**Fig. 4:**
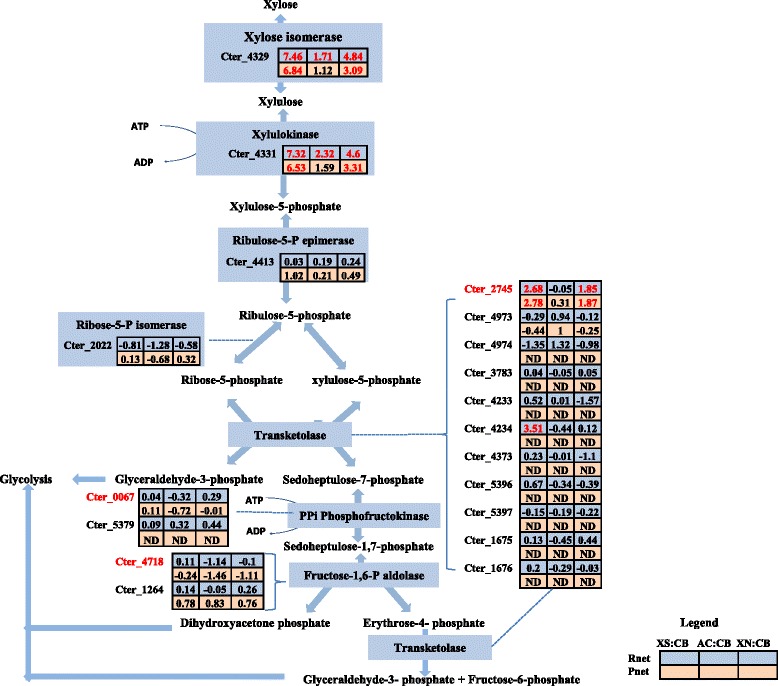
Normalized differential values (Znet-scores) of both the transcriptome (Rnet) and the proteome (Pnet) of reactions involved in the non-oxidative pentose phosphate pathway in *C. termitidis*. In the absence of genes encoding transaldolase, we propose an alternative route for the production of important intermediates using ATP-dependent 6 phosphofructokinase and fructose-1,6-bisphosphate aldolase. Values in red indicate Znet scores of ≥1.65 (outermost 10 %), up regulated in the corresponding substrate with respect to cellobiose and values in green indicate Z-scores of ≤ −1.65 (outermost 10 %), down regulated in the corresponding substrate with respect to cellobiose. Values in black are the innermost 90 %. Locus tags in red indicate high abundance in comparison to paralogs. ND: not detected; XS: xylose; CB: cellobiose; AC: α-cellulose; XN: xylan

The pentose phosphate pathway is important for both the metabolism of 5 carbon sugars, such as xylose, as well as the production of key intermediates namely ribose-5-phosphate and erythrose-4-phosphate, which are required for the synthesis of nucleotides and aromatic amino acids, respectively [33, 34]. Analysis of the genome, transcriptome, and the proteome of *C. termitidis* revealed the presence of all the enzymes needed for the utilization of xylose via the non-oxidative branch of the pentose phosphate pathway, except for a transaldolase enzyme (Fig. 4), which is apparently missing. Xylose isomerase (Cter_4329), which coverts xylose to xylulose, was abundantly expressed under all conditions tested and was detected in the top 10 % of genes and gene products on xylose, xylan, and α-cellulose, with the highest expression observed on xylose. Similarly, xylulose kinase (Cter_4331) was highly up regulated on xylose and xylan and was also among the top 10 % of the genes identified on xylose. In contrast, the genome of *C. thermocellum* does not appear to encode either a xylose isomerase or a xylulokinase, which is consistent with the inability of *C. thermocellum* to grow on pentose sugars [6].

Genome analysis showed that *C. termitidis* encodes seven putative transketolases. Of these, Cter_2745 was highly expressed both in the transcriptome and the proteome under all substrate conditions tested (Additional file 11) and was up regulated on xylose and xylan with respect to cellobiose. Transcripts of Cter_1675-Cter_1676, Cter_3783, Cter_4233-Cter_4234, Cter_4373, Cter_4973-Cter_4974 and Cter_5396-Cter_5397 were also detected albeit at very low levels, suggesting Cter_2745 to be responsible for the inter-conversion to glyceraldehyde-3-phosphate and sedoheptulose −7-phosphate.

Transalodolase, which is known for the degradation of sedoheptulose-7-phosphate to fructose-6-phosphate, for incorporation in to the EMP pathway [34], has not been annotated in *C. termitidis*. Studies in other organisms, have implicated an alternative pathway for sedoheptulose-7-phosphate degradation that does not require a transaldolase [35–37]. In this case, transaldolase would be replaced by a phosphofructokinase, an aldolase, and a transketolase, where PPi/ATP-dependent phoshofructokinase catalyzes the conversion of sedoheptulose-7-phosphate into sedoheptulose-1,7-biphosphate, bifunctional fructose-1,6-bisphosphate aldolase further converts sedoheptulose-1,7-biphosphate to erythrose-4-phosphate and dihydroxyacetone phosphate and, finally, transketolase converts erythrose-4-phosphate into glyceraldehyde-3-phosphate and fructose-6-phosphate, which are eventually directed to the EMP [37]. It is possible that in the absence of a transaldolase, *C. termitidis* channels its intermediates in the pentose phosphate pathway to EMP pathway via this alternative mechanism as indicated in Fig. 4.

#### Formation of pyruvate from phosphoenol pyruvate

**Fig. 5:**
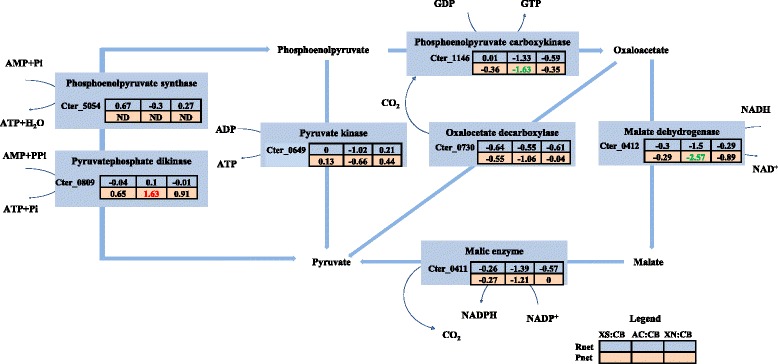
Normalized differential values (Znet-scores) of both the transcriptome (Rnet) and the proteome (Pnet) of reactions involved in the conversion of phosphoenol pyruvate to pyruvate in *C. termitidis*. Values in red indicate Znet scores of ≥1.65 (outermost 10 %), up regulated in the corresponding substrate with respect to cellobiose and values in green indicate Z-scores of ≤ −1.65 (outermost 10 %), down regulated in the corresponding substrate with respect to cellobiose. Values in black are the innermost 90 %. ND: not detected; XS: xylose; CB: cellobiose; AC: α-cellulose; XN: xylan

Phosphoenol pyruvate can be converted to pyruvate, an important control point of the EMP pathway in bacteria, by a number of enzymes through several different routes. PEP is directly converted into pyruvate via an ATP-dependent pyruvate kinase (PPK), reversibly converted via an AMP-dependent pyruvate phosphate dikinase (PPDK) or via an AMP dependent pyruvate water dikinase (phosphoenol pyruvate synthase) [38–41]. In addition, studies in *C. thermocellum* and *C. cellulolyticum* have implicated the malate pathway for conversion of PEP to pyruvate, which utilizes either phosphoenolpyruvate carboxykinase (PEPCK), malate dehydrogenase, and malic enzyme or PEPCK and oxaloacetate decarboxylase (OAADC) for the conversion to pyruvate [6, 7, 42]. In *C. termitidis*, putative genes for enzymes catalyzing all the different routes for pyruvate synthesis from PEP were identified in the current study (presented in Fig. 5; Additional file 11) and were expressed at high levels, except for pyruvate water dikinase Cter_5054, which was expressed at much lower levels and its gene products were not detected under any substrate conditions. The gene products of enzymes in the malate pathway were down regulated on α-cellulose compared to cellobiose and may indicate conversion preference via the alternate route using PPDK, the proteome of which (Cter_0809) showed increased expression on α-cellulose. However, the RNA expression values of PPDK were lower than PPK (Cter_0649) values under all conditions. In *C. thermocellum*, PPDK was similarly found to be up regulated on α-cellulose at stationary phase compared to cellobiose [7]. PPDK plays an anabolic role in gluconeogenesis in some organisms [43, 44], and functions in the catabolic direction in others [40, 45]. As has been shown in *Thermoproteus tenax* [40], the simultaneous presence and expression of PPDK with PPK in *C. termitidis* may represent allosteric differential regulation, thereby providing a way in controlling the inter-conversion of phosphoenolpyruvate and pyruvate and allowing adaptation to different conditions.

#### Pyruvate catabolism and end-product synthesis

**Fig. 6:**
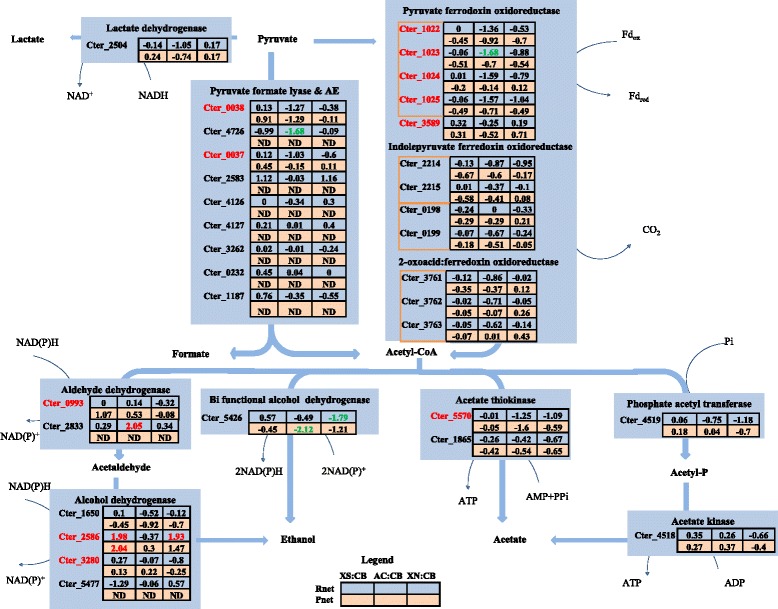
Normalized differential values (Znet-scores) of both the transcriptome (Rnet) and the proteome (Pnet) of reactions involved in the conversion of pyruvate to end products in *C. termitidis*. Values in red indicate Znet scores of ≥1.65 (outermost 10 %), up regulated in the corresponding substrate with respect to cellobiose and values in green indicate Z-scores of ≤ −1.65 (outermost 10 %), down regulated in the corresponding substrate with respect to cellobiose. Values in black are the innermost 90 %. Locus tags in red indicate high abundance in comparison to paralogs. ND: not detected; XS: xylose; CB: cellobiose; AC: α-cellulose; XN: xylan

Growth of *C. termitidis* on both 5 and 6 carbon sugars results in the production of H_2_, CO_2_, ethanol, acetate, formate, and lactate as the major fermentation end-products from pyruvate catabolism. In *C. termitidis,* lactate formation from pyruvate may be catalyzed by the lactate dehydrogenase (LDH) enzyme encoded by Cter_2504 (Fig. 6). While higher amounts of lactate was observed at a lower pH, after late exponential phase, in cultures containing soluble substrates cellobiose and xylose (Additional file 1), LDH RNA transcripts were found in abundance under all substrate conditions (Additional file 11). In terms of relative expression, significant differences were not observed between the substrates tested. High lactate synthesis has been linked to high intracellular concentrations of fructose-1,6-bisphosphate in *Thermoanaerobacter brockii* [46], which is consistent with studies that have shown that LDH enzymes of *C. thermocellum*, *Caldicellulosiruptor saccharolyticus*, *Thermoanaeerobacter ethanolicus* and *Clostridium thermohydrosulfuricans,* to be allosterically activated by fructose-1,6-bisphosphate, pyrophosphate and ATP [47–51]. If this is also true in *C. termitidis*, then higher concentrations of intracellular fructose-1,6-bisphosphate on soluble substrates (xylose and cellobiose, Fig. 3) may be a reason for higher lactate observed. Furthermore, studies in *Thermoanaerobacter wiegelii* Rt8.B1 [52], reported that a decline in intracellular pH during growth resulted in an increase in LDH activity. While the intracellular pH of *C. termitidis* is unknown, a sharp decline in pH under cellobiose and xylose may have additionally contributed to the higher lactate observed.

As in *C. thermocellum*, *C. termitidis* may further convert pyruvate into acetyl coenzyme A, a major branch-point for ethanol and acetate production, via pyruvate ferredoxin oxidoreductase (POR) yielding CO_2_ and reduced ferredoxin, or via pyruvate formate lyase (PFL) yielding formate. According to the annotation, the *C. termitidis* genome encodes one single subunit (Cter_3589), and one multi-subunit POR, which consists of 4 subunits β, α, δ, and γ, encoded by the gene cluster Cter_1022-Cter_1025. While both the gene and the gene products of the 2 PORs identified were expressed abundantly under all conditions tested, Cter-1022-Cter_1025 was down-regulated on α-cellulose with respect to cellobiose (Fig. 6; Additional file 11). In addition, two 2 subunit indole pyruvate ferredoxin oxidoreductases (Cter_2214-Cter_2215 and Cter_0198-Cter_0199) and a multi-subunit 2-oxoacid:ferredoxin oxidoreductase (Cter_3761-Cter_3763) were also detected in this study. Because these enzymes are very similar in sequence and structure to POR, they are sometimes mis-annotated. Indeed, BLAST analysis showed sequence similarity for these genes to POR in other cellulolytic Clostridia, such that these oxidoreductases may putatively play a role in converting pyruvate to acetyl CoA. Nevertheless, the relative abundance of these genes and their products relative to the PORs described above would indicate that Cter_1022-Cter_1025 and Cter_3589 are the primary PORs.

Pyruvate formate lyases, also known as formate acetyl transferases, are the main enzymes involved in formate synthesis during conversion of pyruvate to acetyl-CoA in various organisms including cellulolytic Clostridia [6, 7]. The *C. termitidis* genome encodes for seven putative PFLs (Cter_0038, Cter_1187, Cter_0232, Cter_3262, Cter_4126, Cter_4127 and Cter_4726) and two PFL-activating enzymes (AE) (Cter_0037 and Cter_2583), all of which were transcribed in the current study (Fig. 6). PFL-activating enzymes are important in turning the PFL “on” by acting on the amino acid residues of the PFL catalytic site to form glycyl radical [53]. In the current study, the transcripts of only PFL Cter_0038 and AE Cter_0037 were detected in high abundance under all substrate conditions tested (Additional file 11), and were the only genes with expressed gene products. This suggests that Cter_0037 and Cter_0038 are the main enzymes associated with formate synthesis from pyruvate in *C. termitidis*. While the end-product data shows higher formate under cellobiose and xylose, no significant differences were observed in relative expression profiles for both RNA transcripts and proteomes under all conditions (Fig. 6).

Acetyl-CoA in *C. termitidis* may further be catabolized into ethanol, either directly through a bifunctional acetaldehyde/alcohol dehydrogenase (AdhE encoded by Cter_5426), or indirectly via independent aldehyde dehydrogenases (ALDs, encoded by Cter_0993 and Cter_2833), and a number of independent iron-containing alcohol dehydrogenases (ADHs encoded by Cter_3280, Cter_2586, Cter_1650, and Cter_5477). AdhE (Cter_5426) and ADHs (Cter_3280 and Cter_2586) were observed in high abundance under all conditions tested. While the gene and the gene products of Cter_5426 showed to be up-regulated on cellobiose relative to xylan and α-cellulose, Cter_2586 was up-regulated on xylose and xylan compared to cellobiose (Fig. 6). This may putatively suggest that ethanol production via the two enzyme route was preferred when *C. termitidis* was cultured on 5 carbon sugars, and that ethanol production via AdhE was preferred when *C. termitidis* was cultured on hexose sugars. Cter_2833, Cter_1650 and Cter_5477 were expressed at very low levels and the gene products of Cter_2833 and Cter_5477 were not detected in the proteome, suggesting that these genes do not play any role in ethanol production from acetyl CoA in *C. termitidis* under the substrates tested.

In some anaerobic bacteria, acetate production from acetyl-CoA may proceed directly via acetate thiokinase (ATK, encoded in *C. termitidis* by Cter_5570 and Cter_1865), or indirectly via the co-localized phosphotransacetylase / phosphate acetyl transferase (PTA, encoded by Cter_4519 in *C. termitidis*) and acetate kinase (ACK, encoded by Cter_4518) with the generation of ATP [54–56]. While RNA transcripts and the proteome profiles of enzymes in both pathways were detected under all substrate conditions tested, ACK (Cter_4518) was detected at a much higher level in the proteome, suggesting that acetate production via the indirect route was preferred and that ATK presumably favours the production of acetyl-CoA from acetate [6].

#### Hydrogen generation

In fermentative bacteria such as Clostridia, hydrogenases (H_2_ases) are used for the production of H_2_ to dispose of excess reducing equivalents generated through carbohydrate catabolism [57]. Putative H_2_ases encoded in the *C. termitidis* genome are listed in Table 4. Based on the metal content of the active site, *C. termitidis* H_2_ases can be classified into NiFe H_2_ases and Fe-Fe H_2_ases. In terms of relative expression, no significant differences were observed in the transcriptome and the proteome between the substrate conditions tested.

**Table 4:** Znet-scores (normalized differential values) of *C. termitidis* genes encoding putative hydrogenases

		Znet-scores				Znet-scores	
Locus	Locus-description	R0	P0	R1	P1	R2	P2
NiFe H_2_ase							
Cter_0657	Ni,Fe-hydrogenase III large subunit	0.12	−0.25	−0.68	−1.13	−0.05	−0.74
Cter_3893	Ni,Fe-hydrogenase III large subunit	−0.03	0.71	−1.19	−0.31	0.06	0.25
Cter_3894	Respiratory-chain NADH dehydrogenase, 30 Kd	0.19	0.91	−1.27	0.24	0.3	1.01
Cter_3895	Ni,Fe-hydrogenase III small subunit	−0.02	0.56	−1.32	0.01	0.12	0.52
FeFe H_2_ase							
Cter_4761	Hydrogenase, Fe-only	−0.56		−0.6		−0.91	
Cter_4762	NADH:ubiquinone oxidoreductase, NADH-binding (51 kD)	−0.38	−0.64	−0.41	−0.75	−0.39	−0.23
Cter_4763	Ferredoxin	−0.11		−0.46		1.03	
Cter_4765	NADH-quinone oxidoreductase, E subunit	−1.01		−0.25		−0.19	
**Cter_4848**	Hydrogenase, Fe-only	0.18	−0.24	−0.94	−0.87	−0.98	−0.1
**Cter_4849**	NADH:ubiquinone oxidoreductase, NADH-binding (51 kD)	0.06	−0.25	−1.07	−0.82	−1.2	−0.13
**Cter_4850**	NADH:ubiquinone oxidoreductase 24 kD subunit	0.03	−0.22	−1.44	−0.88	−0.98	−0.24
Cter_2461	Hydrogenase, Fe-only	0.34		0.52		0.23	
Cter_2462	NADH:ubiquinone oxidoreductase, NADH-binding (51 kD)	0.05		−0.19		−0.06	
Cter_2463	NADH:ubiquinone oxidoreductase 24 kD subunit	−0.05		−0.04		−0.11	
Cter_2553	Iron only hydrogenase large subunit	−0.1	0.62	−0.13	0.61	−0.67	0.26
Cter_3528	Iron only hydrogenase large subunit	0.5		0.04		0.25	
Cter_4457	Hydrogenase, Fe-only	0.69		0.81		−0.01	
Cter_4458	NADH:ubiquinone oxidoreductase, NADH-binding (51 kD)	0.19		1.07		0.27	
Cter_4459	NADH:ubiquinone oxidoreductase 24 kD subunit	0.18		0.46		−0.27	

Normalized transcriptomic Z-scores Rnet are represented as: (i) R0: xylose grown cells – cellobiose grown cells; (ii) R1: α-cellulose grown cells – cellobiose grown cells; and (iii) R2: xylan grown cells – cellobiose grown cells. Normalized proteomic Z-scores Pnet are represented as (i) P0: xylose grown cells – cellobiose grown cells; (ii) P1: α-cellulose grown cells – cellobiose grown cells; and (iii) P2: xylan grown cells – cellobiose grown cells. For any protein or RNA transcripts, a negative value represents higher expression on cellobiose, while a positive value represents higher expression in any of the other corresponding substrate. Bold indicates the bifurcating hydrogenase abundantly expressed under all conditions

Of the Fe-Fe H_2_ases, Cter_4848-Cter_4850 was the most highly expressed under all substrate conditions at both the transcription and proteome level. BLAST analyses showed sequence homology with the *C. thermocellum* trimeric bifurcating hydrogenase (encoded by Cthe_0428-Cthe_0430). Bifurcating hydrogenase was initially characterized in *Thermotoga maritama*, which is known to use both NAD(P)H and reduced ferredoxin as substrates, and functions in the production of H_2_ [58]. If this is true in *C. termitidis*, then Cter_4848-Cter_4850 may be the primary H_2_ase responsible for H_2_ production. Cter_4761-Cter_4763 also showed similarity to bifurcating Cthe_0340-Cthe_0342 of *C. thermocellum*. However, the RNA transcripts were expressed at very low levels and only the gene product of Cter_4762 was identified. This is in contrast to the findings in *C. thermocellum*, where both the trimeric bifurcating H_2_ases were detected in high amounts in the proteome [6].

## Conclusion

The ability to grow on and ferment the hydrolysis products of two important lignocellulose derived substrates (cellulose and xylan) to valuable end-products (such as ethanol) makes *C. termitidis* an attractive and potential candidate for biofuel production via consolidated bioprocessing. Understanding the expression of gene and gene products associated with cellulase/hemicellulase synthesis and degradation, and cellular metabolism in conjunction with thermodynamics of end-product formation under different substrates, is important to improving our understanding *C. termitidis* physiology and for identifying engineering targets for improving biomass to biofuel production.

In this study, we conducted RNA seq analysis coupled with 2D HPLC-MS/MS quantitative proteomics for the first time, with *C. termitidis* cultured on simple and complex carbohydrates (cellobiose, xylose, xylan, and α–cellulose) as sole carbon sources to identify and understand the expression of genes and gene products associated with hemi/cellulose degradation, transport and pathways involved in conversion of sugars to end-products. In addition, the present study identified that *C. termitidis* is able to ferment both cellobiose and xylose concomitantly, suggesting the ability to utilize either sugar under mixed substrate conditions.

Of the various cellulosome and non-cellulosome associated CAZymes identified, genes for cellulosomal enzymes and its components were found to be highly expressed on α–cellulose compared to other substrates. This may be due to the fact that, cellulosomes adhere to the cell surface and the solid substrate during growth for maximum nutritional benefits. The lack of an anchor and the availability of readily metabolizable sugars during growth on cellobiose, xylose, and xylan may have contributed to the low expression observed on these substrates. Research using natural biomass as growth substrates may help elucidate this further. In addition, studies on isolated cellulosomes may give insights in to substrate based cellulosome composition and modulation. Furthermore, in terms of CAZyme regulation, we were able to associate a number of glycoside hydrolases with both two component and CcpA regulatory systems.

Through correlation of the transcriptomes and proteomes with the fermentation profiles, we were able to identify the pathways involved in carbon flux in *C. termitidis*. While major differences were not observed in gene and gene product expression for enzymes associated with central metabolism as has been observed in other fermentative organisms [59], xylulokinase and xylose isomerase of the pentose phosphate pathway were found to be highly up- regulated on five carbon sugars compared to hexose sugars. However, to be able to fully understand the regulation of carbon and electron flux, biochemical characterization of associated enzymes, which has not yet been conducted, is necessary. In the presence of multiple paralogs with the same annotation, we were also able to propose which of these were the key genes associated with catabolism based on their expression levels. Nevertheless, this study has enhanced our understanding of the physiology and metabolism of *C. termitidis*, and provides a foundation for future studies on metabolic engineering to optimize biofuel production from natural biomass.

## Methods

### Bacteria and culture conditions

*Clostridium termitidis* CT1112, obtained from American Type Culture Collection (ATCC 51846), was cultured anaerobically at 37 °C in complex 1191 medium [60] containing 2 g/L each of cellobiose, xylose, α-cellulose and beechwood xylan (Sigma-Aldrich Canada) as growth substrates. Batch experiments to investigate the ability of *C. termitidis* to utilize xylose and cellobiose concomitantly were conducted in Balch tubes (Bellco Glass Co.) with a total working volume of 27 mL. 100 g/L sterile, anaerobic cellobiose and xylose were prepared and filter-sterilized into anaerobic, sterile serum bottles (Fisher Scientific) sealed with butyl rubber stopper and aluminium crimp top. These were added to the tubes aseptically to a final concentration of 1 g/L each. For the mixed substrates, cellobiose plus xylose (1 g/L anaerobic cellobiose and 1 g/L anaerobic xylose) were added so that the final concentration was 2 g/L. Fresh cultures were maintained by routinely transferring 10 % (v/v) mid-exponential phase cultures into serum bottles containing fresh 1191 media and various substrates. All chemicals were reagent grade and were obtained from Fischer Scientific unless otherwise stated.

Growth experiments were conducted in 3 independent biological replicates. Cell growth was monitored by spectrophotometry and/or protein concentration (Biochrom, Novaspec II) at 600 nm (OD_600_) for cellobiose, xylose and xylan cultures every four hours (h) and, by total protein concentration for the α-cellulose experiments every 24 h, using a modification of the Bradford method [61]. Briefly, aliquots of each culture were transferred to micro-centrifuge (1.5 mL) tubes and centrifuged to separate the pellets and the supernatants. The pellets were washed with 0.9 % NaCl and re-suspended in 1 mL of 0.2 M NaOH. Samples were incubated at 100 °C in a water bath for 10 minutes (min). Supernatants were collected for Bradford analysis using Bradford’s reagent. Optical densities were measured in 96 well plates at 595 nm using a PowerWave XS multi-plate reader (BIO-TEK, Synergy 4).

### Gas measurement, pH, and end-product analysis

Hydrogen and CO_2_ concentrations were measured using an Agilent 7890A gas chromatograph system (Agilent Technologies Canada Inc. Mississauga, ON), equipped with a Thermal Conductivity Detector (TCD). The pH of each sample was measured using a Sension 2 pH ISE meter (Hach) outfitted with a ThermoOrion triode probe. Soluble end-products and sugar analysis was carried out by high-performance liquid chromatography (Waters Corp., Milford, MA) equipped with a refractive index detector (model 2414) and an ion exclusion column (Aminex HPX-87H; Bio-Rad laboratories, Hercules, CA) using 5 mM sulphuric acid as the mobile phase. Residual cellulose was measured as described previously [18].

Samples for the isolation of total RNA and protein, for RNA seq and proteomic analysis respectively, were collected at exponential phase of growth for each substrate, from two independently replicated experiments. Culture samples were centrifuged in a Sorval (SH BK-3000 rotor) centrifuge at 4750 × g for 15 min at 4 °C to collect pellets. Cell pellets for RNA isolation were submerged in RNA later solution (Life technologies, Burlington, Canada). All cell pellets were stored at −80 °C until further use.

### Total RNA isolation and RNA sequencing

Total RNA from cell pellets was isolated using the ChargeSwitch magnetic bead-based technique for RNA extraction (Life Technologies, Burlington, Canada) according to the manufacturer’s instructions. Samples were treated with DNase to remove contaminating DNA. Total RNA concentrations were measured on NanoDrop spectrophotometer ND-1000 (NanoDrop Technologies, Wilmington, USA), and RNA integrity was assessed using Experion Automated electrophoresis system (Bio RAD, Mississauga, Canada). RNA sequencing was carried out using the Illumina HiSeq 2000 platform by McGill University and Genome Quebec Innovation Center, from high quality RNA samples (RIN > 7).

### Proteomic analysis

Cell pellets were washed thoroughly 3 times with 1X PBS (pH 7.4) and protein was isolated using the filter aided sample preparation method [62].

Proteins were digested in a 1:50 trypsin/protein ratio (Promega, Madison, WI) and the resulting peptides were cleaned as described previously [18] Desalted peptides were stored at −80 °C and lyophilized for subsequent iTRAQ labeling and 2D-HPLC-MS/MS analysis.

Each trypsinized protein sample (~100 μg) was labelled with 4-plex iTRAQ reagent (Applied Biosystems, Foster City, CA, USA), in accordance with manufacturer’s instructions. Samples were labeled as follows: 114: cellobiose (replicate 1); 115: xylose (replicate 1); 116: α-cellulose, (replicate 1); 117: xylan (replicate 1). Samples from biological replicate 2 for each substrate were similarly labeled. Labeling reactions were stopped by diluting with water. Labeled samples were mixed in equal proportions and lyophilized for 2D-HPLC-MS/MS.

Mixed iTRAQ labelled peptides were spiked with a mixture of six internal standard peptides [63], fractionated on the Agilent 1100 Series HPLC system using the C18 X-Terra column and eluted as previously described [18]. Peptide fractions collected were concatenated as described by Dwivedi et al. [64] into a total of 26 fractions. Fractions were lyophilized and stored at −80 °C until needed. Each fraction was re-suspended in 100 μL of 0.1 % formic acid for second (2nd) dimension separation.

On-line 2nd dimension separation was carried out on a splitless nanoflow Tempo LC system (Eksigent, Dublin, CA). Twenty (20) μL of sample was injected via a PepMap100 trap column (0.3 mm × 5 mm, 5 μm, 100 Å; Dionex Corporation, Sunnyvale, CA, USA) and a 100 μm × 150 mm analytical column packed with 5 μm Luna C18(2) (Phenomenex, Torrance, CA). Both eluents A (2 % acetonitrile in water) and B (98 % acetonitrile) contained 0.1 % formic acid as ion-pairing modifier. A 0.35 % acetonitrile/min linear gradient (0–35 % B) was used for peptide elution, providing a total of 2 h run time per fraction. TripleTOF 5600 mass spectrometer (Applied Biosystems, Foster City, CA) was used in standard MS/MS data-dependent acquisition mode with a nano-electrospray ionization source. The spectrometer was set to perform data acquisition in the positive ion mode, with a selected mass range of m/z 400 to 1500 for 1 second (s). This was followed by three MS/MS measurements on the most intense parent ions (80 counts/s threshold, +2 to +4 charge state, and m/z 100 to 1500 mass range for MS/MS), using the manufacturer's ‘smart exit’ and built in iTRAQ settings.

### Protein and RNA identification and statistical analysis

Reads from RNAseq and the peptide MS/MS spectra from proteomic analysis were identified by mapping to the IMG/ER version of the *C. termitidis* annotation of the genome available at DDBJ/EMBL/GenBank under the accession AORV00000000. For proteins, raw WIFF spectrum collection files were converted into Mascot Generic Format (MGF) using the Mascot.dll script bundled with Analyst QS2.0 [65]. These collections of CID spectra were analyzed using our in-house graphic processor unit (GPU)-based peptide identification engine [66] and X!tandem with the same search settings. A protein level false positive rate of 0.4% and a false positive peptide count of approximately 500 out of 23,039 uniquely assigned was reported. Settings used were: tryptic peptides, up to one missed cleavage; 100 ppm and 0.4 Da mass tolerances for parent and fragment ions respectively, and a constant PTM of carbamidomethylation on cysteine (C + 57.021). Candidate peptide masses and CID parent masses must fall within a fixed tolerance, typically 20 PPM for the TripleTOF 5600 mass spectrometer. Transcriptomic analysis (RNA seq) was performed using an in-house alignment engine that enumerates exact 100mer alignments against their source genes. The entire RNA seq dataset has been deposited in NCBI’s Gene Expression Omnibus (GEO, [67]) database under accession number GSE66125 (http://www.ncbi.nlm.nih.gov/geo/query/acc.cgi?acc=GSE66125).

We have developed a simple platform for mapping multi-omic expression values into a common analysis matrix using our in-house lobe/UNITY analysis platform [68]. Columns contain log2 expression values for a given experiment, while rows encode the source proteins/genes, their annotation descriptions, and each gene’s mapping into four “higher-order variables” (HOVs) extracted from the IMG-ER “Export Gene Information” function [69]. These HOVs, which include METACYC pathways, enzyme class (EC) numbers, clusters of orthologous group (COG) letters, and KEGG pathway modules, were selected to both overlap somewhat and provide useful degrees of biological granularity. Our analysis uses a set of relatively simple scatterplot, counting, mapping, transformation, difference and filtering functions applied to this matrix. Prior to analysis, expression data from RNAseq and proteomics is subjected to some simple filtering: RNAseq values must have at least two alignment counts, and proteins must have at least two peptides with expectation values of log(e) < −1.5 each.

Expression values as total ion counts (TIC) were the log2 value of the sum of their member peptide iTRAQ reporter-ion intensities (CID fragments) for proteomics and, log2 of the sum of 100-mer alignment elements per gene for transcriptomics for simplified differential analysis and comparison. Relative expression levels between growth conditions for RNA transcripts were computed using a simple transformation, as described by Verbeke et al. [59], which combines difference measurements into unified expression notations: R, based on RNA level Z-scores and measured in units of standard deviation. For each comparison, four different measurements were determined, which incorporate the intra replicate (Z0 = B_X_–A_X_, Z1 = B_Y_–A_Y_), as well as the cross state (Z2 = A_X_ –A_Y_; Z3 = B_X_–B_Y_) variability, where A and B represent two biological replicates and X and Y represent different substrates. In the case of 2D-HPLC MS/MS, due to the large differences in the number of CID spectra collected between biological replicates (278,000 versus 146,000) and corresponding differences in peptide and protein identification and quantitation, we opted to conduct analysis using only the larger run set. The validity of the current proteomic data was however confirmed by comparing it to a previous iTRAQ run on α-cellulose and cellobiose [18] (Additional file 12). Therefore only the cross state difference measurements: P, were used for the analysis in this study. Although this earlier data was collected using a previous generation of instrumentation, the ~ 1400 proteins common between these experiments gave log2 intensity correlations of *R*^2^ ~ 0.7 in both states (alpha cellulose and cellobiose). Differences between the alpha cellulose and cellobiose protein expression across these two experiments similarly correlated.

For both RNA seq and proteomics, data from xylose, xylan and α-cellulose was measured against cellobiose. The resulting difference populations were each normalized to a mean of zero and a standard deviation of 1 and subjected to a simple algorithm for merging them into a single normalized expression value, Rnet and Pnet, for transcriptomics and proteomics respectively, which were used in subsequent analysis. This final differential expression distribution is roughly Gaussian, with the outermost 33 % of the population with Z-scores of > 1, outermost 10 % of the population having absolute-value Z-scores >1.65 and the outermost 5 % of the population having absolute-value Z-scores >1.96. For the current work, normalized transcriptomic values Rnet are represented as follows: (i) R0 = xylose grown cells – cellobiose grown cells; (ii) R1 = α-cellulose grown cells – cellobiose grown cells; and (iii) R2 = xylan grown cells – cellobiose grown cells. Similarly, normalized proteomic values Pnet are represented as follows: (i) P0 = xylose grown cells – cellobiose grown cells; (ii) P1 = α-cellulose grown cells – cellobiose grown cells; and (iii) P2 = xylan grown cells – cellobiose grown cells. Therefore, for any protein or RNA, a negative value represents higher expression on cellobiose, while a positive value represents higher expression in any of the other corresponding substrate. Since Z-scores are normalized differential expression values between growth states, in units of standard deviation for their respective populations, a Z-score magnitude cutoff of approximately ≥ 1.65, (representing approximately the outermost ten percent of the difference population), provides a good compromise between stringency and differential expression exploration.

### Data validation

RNA and Protein expression data correlation under each experimental condition was carried out by plotting the Log2 expression values of transcriptomic and proteomic data (Additional file 4). The dynamic range of Log2 expression values for RNAseq and proteomic analyses was > 4 and > 10 respectively. Furthermore, a simple function was developed to evaluate the statistical significance of a two-state two-replicate dataset, on a gene-by-gene basis. This function computes an overall measurement of the system quality as the ratio of the mean of the standard deviations of the cross state and intra-replicate populations. This is known as the system to noise (S/N) ratio. On an individual gene level, the S/N is the ratio of the vector magnitudes across states and intra-replicate normalized values, scaled by the overall system S/N values. All genes with a S/N > 2.8 were found to have a False discovery rate of 10 % or less, which was computed through a simple Monte-Carlo Model. This function allowed reliable access to smaller differential expression values across states, provided the variation in corresponding intra-replicate measurements were sufficiently small.

### Genome analysis

Unless specified, BLASTP search tools with default settings in IMG-ER were used to assess similarity with other bacteria [69]. Putative catabolite responsive elements (*Cre* sequences) in the *C. termitidis* genome were searched for with an ad hoc perl script using the reported consensus sequence from *Bacillus subtilis* [70] as a query.

## Additional files

Additional file 1: End-product synthesis patterns in cultures of *C. termitidis* containing 2 g/L α-cellulose, cellobiose, xylan, and xylose. Gas production (A) and soluble end-products (B) in α-cellulose cultures; Gas production (C) and soluble end-products (D) in cellobiose cultures; Gas production (E) and soluble end-products (F) in xylan culture; Gas production (G) and soluble end-products (H) in xylose cultures Bars above and below the means represent standard deviation between replicates. (PDF 253 kb) Additional file 2: Linear regression analysis of the sum of log2 100-mer reads per gene, between biological replicates. Values given for replicates grown on 2 g/L each α-cellulose (A); cellobiose (B); xylan (C); and xylose (D). Values observed for biological replicate 1 are plotted on the x-axis, while the corresponding values for biological replicate 2 are plotted on the y-axis. (PDF 1010 kb) Additional file 3: Scatter plots showing differential RNA seq Z-scores between replicates and across state (substrates). Cross state analysis was carried out against cellobiose in all cases. AC: α-cellulose; CB: cellobiose. Values plotted along axis are from both biological replicates. (PDF 917 kb) Additional file 4: Correlation of log2 expression values of transcriptomic and proteomic under the four experimental conditions. A: Cellobiose; B: Xylose; C: α-cellulose; D: Xylan (PDF 426 kb) Additional file 5: Overall biological signal to systematic noise ratio of RNAseq analyses under four experimental conditions. AC: α-cellulose; CB: cellobiose; Rep: replicate (PDF 85 kb) Additional file 6: Expression values and Znet values of genes and gene products identified in *C. termitidis* cultured under various substrate conditions. Normalized proteomic Z-scores Pnet are represented as: (i) P0: xylose grown cells – cellobiose grown cells; (ii) P1: α-cellulose grown cells – cellobiose grown cells; and (iii) P2: xylan grown cells – cellobiose grown cells. Normalized transcriptomic Z-scores Rnet are represented as: (i) R0: xylose grown cells – cellobiose grown cells; (ii) R1: α-cellulose grown cells – cellobiose grown cells; and (iii) R2: xylan grown cells – cellobiose grown cells. For any protein or RNA transcripts, a negative value represents higher expression on cellobiose, while a positive value represents higher expression in any of the other corresponding substrate. Signal to Noise ratios (S/N) of individual genes under two comparing conditions are given. S/N0: xylose grown cells vs cellobiose grown cells; S/N1: α-cellulose grown cells – cellobiose grown cells; S/N2: xylan grown cells – cellobiose grown cells. Genes with a S/N value 2.8 or greater have a false discovery rate (FDR) 10 % or less. CB, Cellobiose; AC, α-cellulose; Numbers in brackets indicate biological replicate. (XLSX 817 kb) Additional file 7: Znet scores of LacI transcriptional regulators and Histidine containing proteins in *C. termitidis*. Normalized proteomic Z-scores Pnet are represented as: (i) P0: xylose grown cells – cellobiose grown cells; (ii) P1: α-cellulose grown cells – cellobiose grown cells; and (iii) P2: xylan grown cells – cellobiose grown cells. Normalized transcriptomic Z-scores Rnet are represented as: (i) R0: xylose grown cells – cellobiose grown cells; (ii) R1: α-cellulose grown cells – cellobiose grown cells; and (iii) R2: xylan grown cells – cellobiose grown cells. Highlighted cells represent Znet-value threshold for up and down regulation of ± 1.65 (representing the outermost 10 % of the data set). (XLSX 11 kb) Additional file 8: Potential *cre* sequences identified within the *C. termitidis* CT1112 genome (XLSX 19 kb) Additional file 9: Highly expressed ABC type sugar transporters identified in the transcriptome and proteome of *C. termitidis.* Normalized proteomic Z-scores Pnet are represented as: (i) P0: xylose grown cells – cellobiose grown cells; (ii) P1: α-cellulose grown cells – cellobiose grown cells; and (iii) P2: xylan grown cells – cellobiose grown cells. Normalized transcriptomic Z-scores Rnet are represented as: (i) R0: xylose grown cells – cellobiose grown cells; (ii) R1: α-cellulose grown cells – cellobiose grown cells; and (iii) R2: xylan grown cells – cellobiose grown cells. Highlighted cells represent Znet-value threshold for up and down regulation of ± 1.65 (representing the outermost 10 % of the data set). Up-regulation in the corresponding substrate with respect to cellobiose are highlighted in red, while down-regulation in the corresponding substrate with respect to cellobiose are highlighted in green. (XLSX 11 kb) Additional file 10: Gas production and soluble end-product synthesis patterns of *C.termitidis* cultured on 1 g/L cellobiose, 1 g/L xylose, and 1 g/L cellobiose plus 1 g/L xylose. Gas production (A) in 1 g/L cellobiose cultures; Gas production (B) in 1 g/L xylose cultures; Gas production (C) in 1 g/L cellobiose plus 1 g/L xylose cultures; soluble end products (D) in 1 g/L cellobiose cultures; soluble end products (E) in 1 g/L xylose cultures; soluble end products (F) in 1 g/L cellobiose plus 1 g/L xylose culture. Bars above and below the means represent standard deviation between replicates. (PDF 150 kb) Additional file 11: RNA seq and Proteomics expression values of enzymes identified in *C. termitidis* carbon metabolism pathways. CB: cellobiose; AC: α –cellulose. Numbers in brackets indicate replicate samples (XLSX 18 kb) Additional file 12: Proteomic expression values and Znet scores of previously run iTRAQ on 2 g/L each of α-cellulose and cellobiose (XLSX 208 kb)

## Abbreviations

2D-HPLC MS/MS: two dimensions high profile chromatography mass spectrometry
ABC transporters: ATP binding cassette transporters
ACK: acetate kinase
AdhE: bifunctional acetaldehyde/alcohol dehydrogenase
ADHs: alcohol dehydrogenases
ALDs: aldehyde dehydrogenases
ATK: acetate thiokinase
ATP: adenosine triphosphate
BLAST: basic local alignment search tool
CAZymes: carbohydrate active enzymes
CBP: consolidated bioprocessing
CCPA: carbon catabolite protein A
CCR: carbon catabolite repression
CID: collision induced dissociation
CO_2_: carbon-dioxide
COGs: cluster of orthologous groups
EMP: Embden Meyerhof parnas pathway
FN3: fibronectin type 3 domain
H_2_: hydrogen
HOVs: higher order variables
IMG/ER: integrated microbial genomics expert review
iTRAQ: isobaric tags for relative and absolute quantitation
KEGG: Kyoto Encyclopedia of Genes and Genomes
LDH: lactate dehydrogenase
MetaCyc: metabolic pathways and enzymes database
NiFe H_2_ases: nickel iron hydrogenases
OAADC: oxaloacetate decarboxylase
PEP: phosphoenol pyruvate
PEPCK: phosphoenolpyruvate carboxykinase
PFL: pyruvate formate lyase
POR: pyruvate ferredoxin oxidoreductase
PPDK: AMP-dependent pyruvate phosphate dikinase
PPi: inorganic pyrophospahete
PPK: ATP-dependent pyruvate kinase
PTA: phosphate acetyl transferase
RIN: RNA integrity number
RNA seq: RNA sequencing
TCS: two component regulatory system
TIC: total ion current

## References

[1] Demain AL, Newcomb M, Wu JHD (2005). Cellulase clostridia, and ethanol. Microbiol Mol Biol Rev.

[2] Lynd LR, Weimer PJ, Van Zyl WH, Isak S, Pretorius IS (2002). Microbial cellulose utilization: fundamentals and biotechnology. Microbiol Mol Biol Rev.

[3] Ulrich-Merzenich G, Zeitler H, Jobst D, Panek D, Vetter H, Wagner H (2007). Application of the “-Omic-” technologies in phytomedicine. Phytomedicine.

[4] Wei H, Fu Y, Magnusson L, Baker JO, Maness P-C, Xu Q, Yang S, Bowersox A, Bogorad I, Wang W (2014). Comparison of transcriptional profiles of Clostridium thermocellum grown on cellobiose and pretreated yellow poplar using RNA-Seq. Front Microbiol.

[5] Wilson CM, Yang S, Rodriguez M, Ma Q, Johnson CM, Dice L, Xu Y, Brown SD (2013). Clostridium thermocellum transcriptomic profiles after exposure to furfural or heat stress. Biotechnol Biofuels.

[6] Rydzak T, McQueen PD, Krokhin O, Spicer V, Ezzati P, Dwivedi RC, Shamshurin D, Levin DB, Wilkins JA, Sparling R (2012). Proteomic analysis of Clostridium thermocellum core metabolism: relative protein expression profiles and growth phase-dependent changes in protein expression. BMC Microbiol.

[7] Raman B, McKeown CK, Rodriguez M, Brown SD, Mielenz JR (2011). Transcriptomic analysis of Clostridium thermocellum ATCC 27405 cellulose fermentation. BMC Microbiol.

[8] Stevenson DM, Weimer PJ (2005). Expression of 17 genes in Clostridium thermocellum ATCC 27405 during fermentation of cellulose or cellobiose in continuous culture. Appl Environ Microbiol.

[9] Raman B, Pan C, Hurst GB, Rodriguez M, McKeown CK, Lankford PK (2009). Impact of pretreated Switchgrass and biomass carbohydrates on Clostridium thermocellum ATCC 27405 cellulosome composition: a quantitative proteomic analysis. PLoS One.

[10] Xu C, Huang R, Teng L, Wang D, Hemme CL, Borovok I, He Q, Lamed R, Bayer EA, Zhou J (2013). Structure and regulation of the cellulose degradome in *Clostridium cellulolyticum*. Biotechnol Biofuels.

[11] Ramachandran U, Wrana N, Cicek N, Sparling R, Levin DB (2008). Hydrogen production and end-product synthesis p1. Ramachandran U, Wrana N, Cicek N, Sparling R, Levin DB: Hydrogen production and end-product synthesis patterns by *Clostridium termitidis* strain CT1112 in batch fermentation cultures with cellobiose. Int J Hydrogen Energy.

[12] Hethener P, Brauman A, Garcia J-L (1992). *Clostridium termitidis* sp. nov., a Cellulolytic Bacterium from the Gut of the Wood-feeding Termite, *Nasutitermes lujae*. Syst Appl Microbiol.

[13] Munir RI, Schellenberg J, Henrissat B, Verbeke TJ, Sparling R, Levin DB (2014). Comparative analysis of carbohydrate active enzymes in Clostridium termitidis CT1112 reveals complex carbohydrate degradation ability. PLoS One.

[14] Bayer EA, Belaich J-P, Shoham Y, Lamed R (2004). The cellulosomes: multienzyme machines for degradation of plant cell wall polysaccharides. Annu Rev Microbiol.

[15] Doi RH, Kosugi A (2004). Cellulosomes: plant-cell-wall-degrading enzyme complexes. Nat Rev Microbiol.

[16] Tamaru Y (2001). The Clostridium cellulovorans cellulosome: an enzyme complex with plant cell wall degrading activity. Chem Rec.

[17] Schwarz WH (2001). The cellulosome and cellulose degradation by anaerobic bacteria. Appl Microbiol Biotechnol.

[18] Munir RI, Spicer V, Shamshurin D, Krokhin OV, Wilkins J, Ramachandran U, Sparling R, Levin DB (2015). Quantitative proteomic analysis of the cellulolytic system of Clostridium termitidis CT1112 reveals distinct protein expression profiles upon growth on α-cellulose and cellobiose. J Proteomics.

[19] Lynd LR, van Zyl WH, McBride JE, Laser M (2005). Consolidated bioprocessing of cellulosic biomass: an update. Curr Opin Biotechnol.

[20] Zverlov VV, Kellermann J, Schwarz WH (2005). Functional subgenomics of Clostridium thermocellum cellulosomal genes: Identification of the major catalytic components in the extracellular complex and detection of three new enzymes. Proteomics.

[21] Gold ND, Martin VJJ (2007). Global view of the Clostridium thermocellum cellulosome revealed by quantitative proteomic analysis. J Bacteriol.

[22] De Philip P, Lignon S, Tardif C, Page S (2010). Modulation of cellulosome composition in Clostridium cellulolyticum: adaptation to the polysaccharide environment revealed by proteomic and carbohydrate- active enzyme analyses. Proteomics.

[23] Tamaru Y, Miyake H, Kuroda K, Nakanishi A, Matsushima C, Doi RH, Ueda M (2011). Comparison of the mesophilic cellulosome-producing Clostridium cellulovorans genome with other cellulosome-related clostridial genomes. Microb Biotechnol.

[24] Bayer E, Lamed R, White B, Flint H (2008). From cellulosomes to cellulosomics. Chem Rec (New York, NY).

[25] Bagnara-Tardif C, Gaudin C, Belaich A, Hoest P, Citard T, Belaich JP (1992). Sequence analysis of a gene cluster encoding cellulases from Clostridium cellulolyticum. Gene.

[26] Kataeva IA, Seidel RD, Shah A, West LT, Li X-L, Ljungdahl LG (2002). The fibronectin type 3-like repeat from the Clostridium thermocellum cellobiohydrolase CbhA promotes hydrolysis of cellulose by modifying its surface. Appl Environ Microbiol.

[27] Fujita Y (2009). Carbon catabolite control of the metabolic network in Bacillus subtilis. Biosci Biotechnol Biochem.

[28] Warner JB, Lolkema JS (2003). CcpA-dependent carbon catabolite repression in bacteria. Microbiol Mol Biol Rev.

[29] Abdou L, Boileau C, De Philip P, Pagès S, Fiérobe HP, Tardif C (2008). Transcriptional regulation of the Clostridium cellulolyticum cip-cel operon: a complex mechanism involving a catabolite-responsive element. J Bacteriol.

[30] Stock AM, Robinson VL, Goudreau PN (2000). Two-component signal transduction. Annu Rev Biochem.

[31] Celik H, Blouzard JC, Voigt B, Becher D, Trotter V, Fierobe HP, Tardif C, Pagès S, de Philip P (2013). A two-component system (XydS/R) controls the expression of genes encoding CBM6-containing proteins in response to straw in clostridium cellulolyticum. PLoS One.

[32] Schurig H, Beaucamp N, Ostendorp R, Jaenicke R, Adler E, Knowles JR (1995). Phosphoglycerate kinase and triosephosphate isomerase from the hyperthermophilic bacterium Thermotoga maritima form a covalent bifunctional enzyme complex. EMBO J.

[33] Sprenger G a (1995). Genetics of pentose-phosphate pathway enzymes of Escherichia coli K-12. Arch Microbiol.

[34] Zeikus JG (1980). Chemical and fuel production by anaerobic bacteria. Annu Rev Microbiol.

[35] Flechner A, Gross W, Martin WF, Schnarrenberger C (1999). Chloroplast class I and class II aldolases are bifunctional for fructose-1,6-biphosphate and sedoheptulose-1,7-biphosphate cleavage in the Calvin cycle. FEBS Lett.

[36] Mertens E, De Jonckheere J, Van Schaftingen E (1993). Pyrophosphate-dependent phosphofructokinase from the amoeba Naegleria fowleri, an AMP-sensitive enzyme. Biochem J.

[37] Susskind BM, Warren LG, Reeves RE (1982). A pathway for the interconversion of hexose and pentose in the parasitic amoeba Entamoeba histolytica. Biochem J.

[38] Carere CR, Rydzak T, Verbeke TJ, Cicek N, Levin DB, Sparling R (2012). Linking genome content to biofuel production yields: a meta-analysis of major catabolic pathways among select H2 and ethanol-producing bacteria. BMC Microbiol.

[39] Sparling R, Carere C, Rydzak T, Schellenberg J, Levin DB, Azbar N, Levin DB (2012). Comparative genomics and bioenergetics of dark fermentation (Chapter 10). State of the art and progress in production of biohydrogen.

[40] Tjaden B, Plagens A, Dörr C, Siebers B, Hensel R (2006). Phosphoenolpyruvate synthetase and pyruvate, phosphate dikinase of Thermoproteus tenax: Key pieces in the puzzle of archaeal carbohydrate metabolism. Mol Microbiol.

[41] Lengler WJ, Drews J, Schlegel GH (1999). Biology of prokaryotes.

[42] Li Y, Tschaplinski TJ, Engle NL, Hamilton CY, Rodriguez M, Liao JC, Schadt CW, Guss AM, Yang Y, Graham DE (2012). Combined inactivation of the Clostridium cellulolyticum lactate and malate dehydrogenase genes substantially increases ethanol yield from cellulose and switchgrass fermentations. Biotechnol Biofuels.

[43] Rodriguez-Contreras D, Hamilton N (2014). Gluconeogenesis in Leishmania Mexicana. Contribution of glycerol kinase, phosphoenolpyruvate carboxykinase, and pyruvate phosphate dikinase. J Biol Chem.

[44] Benziman M, Eizen N (1971). Pyruvate-phosphate dikinase and the control of gluconeogenesis in Acetobacter xylinum. J Biol Chem.

[45] Reeves RE, Menzies R a, Hsu DS (1968). The pyruvate-phosphate dikinase reaction. The fate of phosphate and the equilibrium. J Biol Chem.

[46] Ben-Bassat A, Lamed R, Zeikus JG (1981). Ethanol production by thermophilic bacteria: metabolic control of end product formation in Thermoanaerobium brockii. J Bacteriol.

[47] Yang S, Giannone RJ, Dice L, Yang ZK, Engle NL, Tschaplinski TJ, Hettich RL, Brown SD (2012). Clostridium thermocellum ATCC27405 transcriptomic, metabolomic and proteomic profiles after ethanol stress. BMC Genomics.

[48] Willquist K, van Niel EWJ (2010). Lactate formation in Caldicellulosiruptor saccharolyticus is regulated by the energy carriers pyrophosphate and ATP. Metab Eng.

[49] Zhou Q, Shao W-L (2010). Molecular genetic characterization of the thermostable L-lactate dehydrogenase gene (ldhL) of Thermoanaerobacter ethanolicus JW200 and biochemical characterization of the enzyme. Biochemistry (Mosc).

[50] Lovitt RW, Shen GJ, Zeikus JG (1988). Ethanol production by thermophilic bacteria: biochemical basis for ethanol and hydrogen tolerance in Clostridium thermohydrosulfuricum. J Bacteriol.

[51] Özkan M, Yllmaz EI, Lynd LR, Özcengiz G (2004). Cloning and expression of the Clostridium thermocellum L-lactate dehydrogenase gene in Escherichia coli and enzyme characterization. Can J Microbiol.

[52] Cook GM (2000). The intracellular pH of the thermophilic bacterium Thermoanaerobacter wiegelii during growth and production of fermentation acids. Extremophiles.

[53] Becker A, Kabsch W (2002). X-ray structure of pyruvate formate-lyase in complex with pyruvate and CoA. How the enzyme uses the Cys-418 thiyl radical for pyruvate cleavage. J Biol Chem.

[54] Lamed R, Zeikus JG (1980). Ethanol production by thermophilic bacteria: relationship between fermentation product yields of and catabolic enzyme activities in Clostridium thermocellum and Thermoanaerobium brockii. J Bacteriol.

[55] Lin WR, Lee CC, Hsu JJ, Hamel JF, Demain AL (1998). Properties of acetate kinase activity in Clostridium thermocellum cell extracts. Appl Biochem Biotechnol.

[56] Melville SB, Michel TA, Macy JM (1988). Pathway and sites for energy conservation in the metabolism of glucose by selenomonas ruminantium. J Bacteriol.

[57] Vignais PM, Billoud B, Meyer J (2001). Classification and phylogeny of hydrogenases. FEMS Microbiol Rev.

[58] Schut GJ, Adams MWW (2009). The iron-hydrogenase of Thermotoga maritima utilizes ferredoxin and NADH synergistically: a new perspective on anaerobic hydrogen production. J Bacteriol.

[59] Verbeke TJ, Spicer V, Krokhin OV, Zhang X, Schellenberg JJ, Fristensky B, Wilkins JA, Levin DB, Sparling R (2014). Thermoanaerobacter thermohydrosulfuricus WC1 shows protein complement stability during fermentation of Key lignocellulose-derived substrates. Appl Environ Microbiol.

[60] Islam R, Cicek N, Sparling R, Levin D (2006). Effect of substrate loading on hydrogen production during anaerobic fermentation by Clostridium thermocellum 27405. Appl Microbiol Biotechnol.

[61] Bradford MM (1976). A rapid and sensitive method for the quantitation of microgram quantities of protein utilizing the principle of protein-dye binding. Anal Biochem.

[62] Wisniewski JR, Zougman A, Nagaraj N, Mann M (2009). Universal sample preparation method for proteome analysis. Nat Methods.

[63] Krokhin OV, Spicer V (2009). Peptide retention standards and hydrophobicity indexes in reversed-phase high-performance liquid chromatography of peptides. Anal Chem.

[64] Dwivedi RC, Spicer V, Harder M, Antonovici M, Ens W, Standing KG, Wilkins JA, Krokhin OV (2008). Practical implementation of 2D HPLC scheme with accurate peptide retention prediction in both dimensions for high-throughput bottom-up proteomics. Anal Chem.

[65] Perkins DN, Pappin DJC, Creasy DM, Cottrell JS (1999). Probability-based protein identification by searching sequence databases using mass spectrometry data. Electrophoresis.

[66] McQueen P, Spicer V, Rydzak T, Sparling R, Levin D, Wilkins JA, Krokhin O (2012). Information-dependent LC-MS/MS acquisition with exclusion lists potentially generated on-the-fly: case study using a whole cell digest of Clostridium thermocellum. Proteomics.

[67] Edgar R, Domrachev M, Lash AE (2002). Gene expression omnibus: NCBI gene expression and hybridization array data repository. Nucleic Acids Res.

[68] McQueen P, Spicer V, Schellenberg J, Krokhin O, Sparling R, Levin D, Wilkins JA (2015). Whole cell, label free protein quantitation with data independent acquisition: quantitation at the MS2 level. Proteomics.

[69] Markowitz VM, Mavromatis K, Ivanova NN, Chen I-MA, Chu K, Kyrpides NC (2009). IMG ER: a system for microbial genome annotation expert review and curation. Bioinformatics.

[70] Miwa Y, Nakata A, Ogiwara A, Yamamoto M, Fujita Y (2000). Evaluation and characterization of catabolite-responsive elements (cre) of Bacillus subtilis. Nucleic Acids Res.

