# Supplementary figures and images for: Transcriptomic and proteomic analyses of core metabolism in Clostridium termitidis CT1112 during growth on α-cellulose, xylan, cellobiose and xylose
Source: BMC Microbiol. 2016 May 23;16:91. doi: 10.1186/s12866-016-0711-x (PMC4877739; doi:10.1186/s12866-016-0711-x)

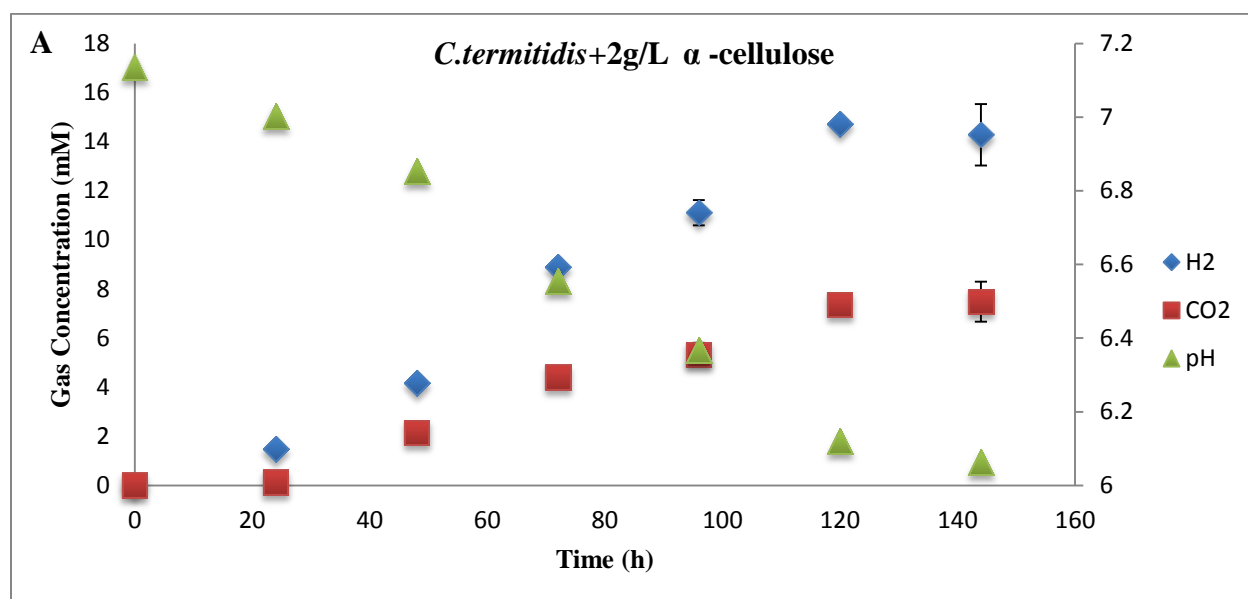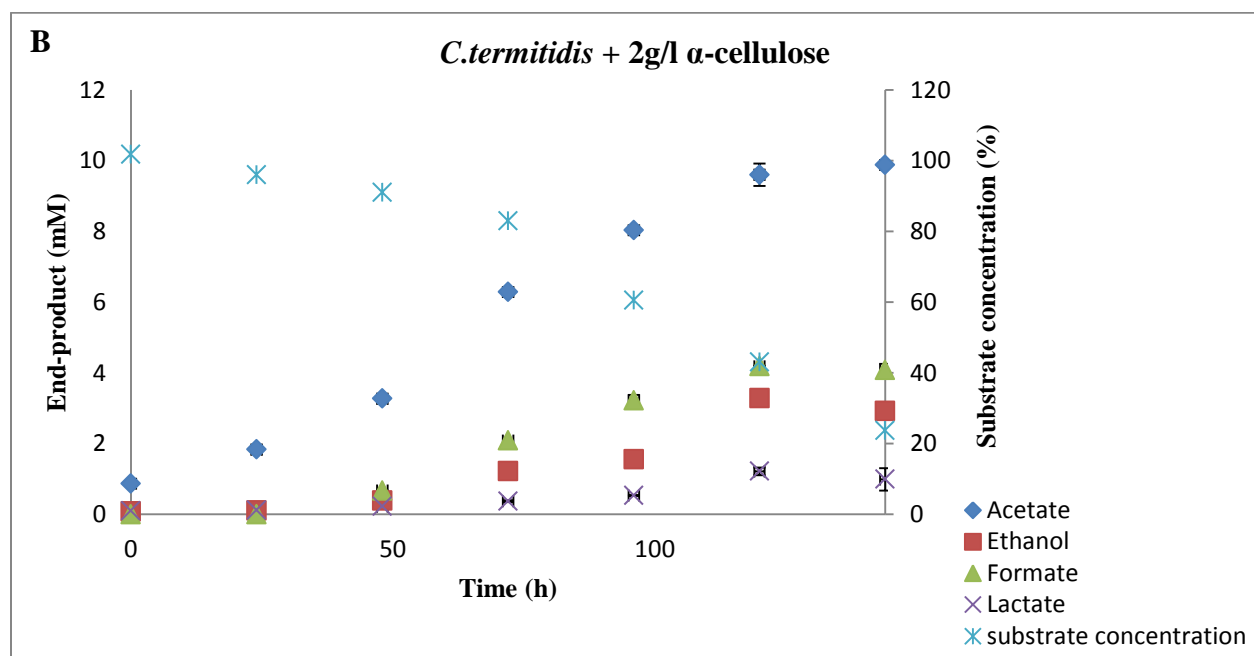

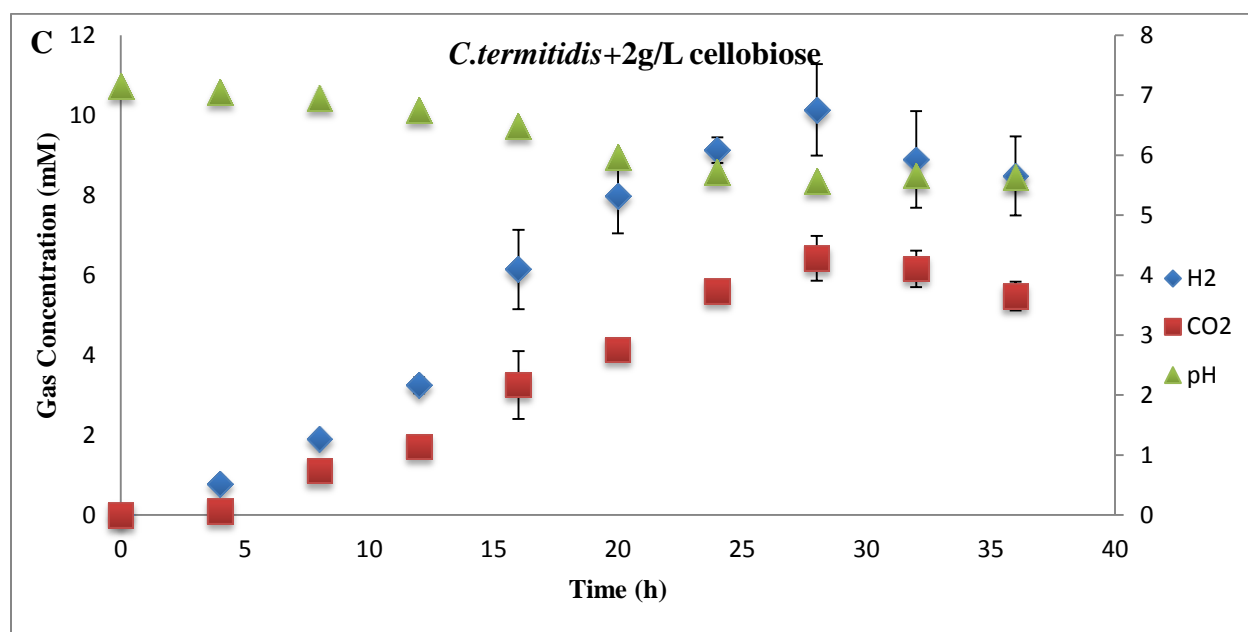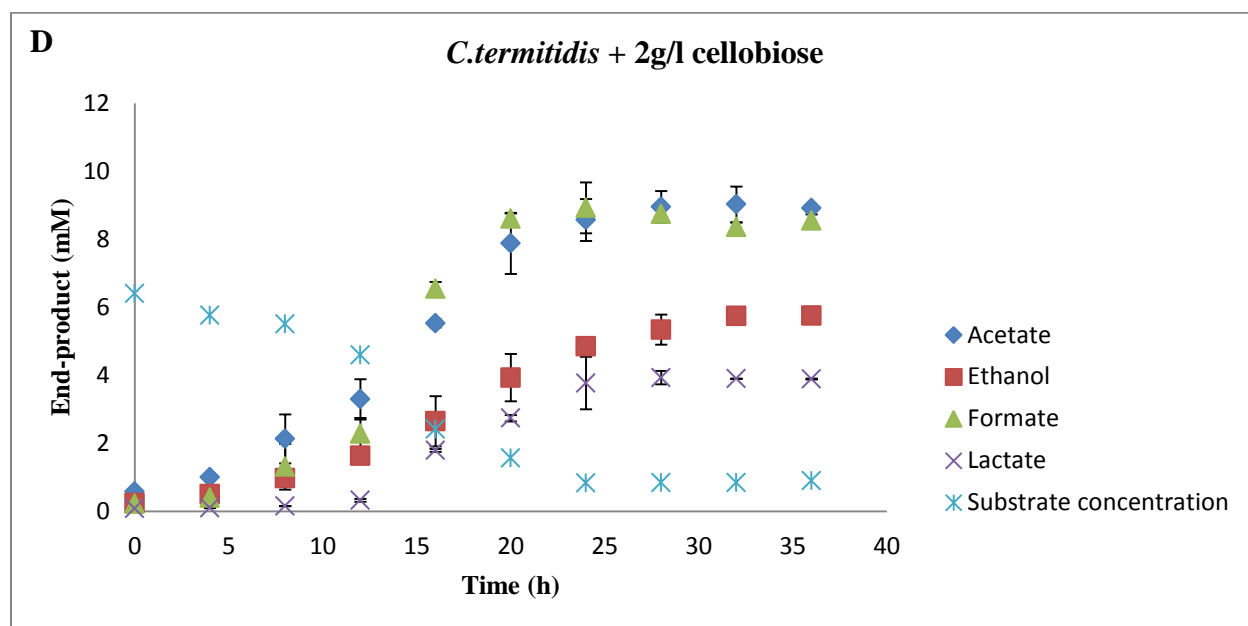

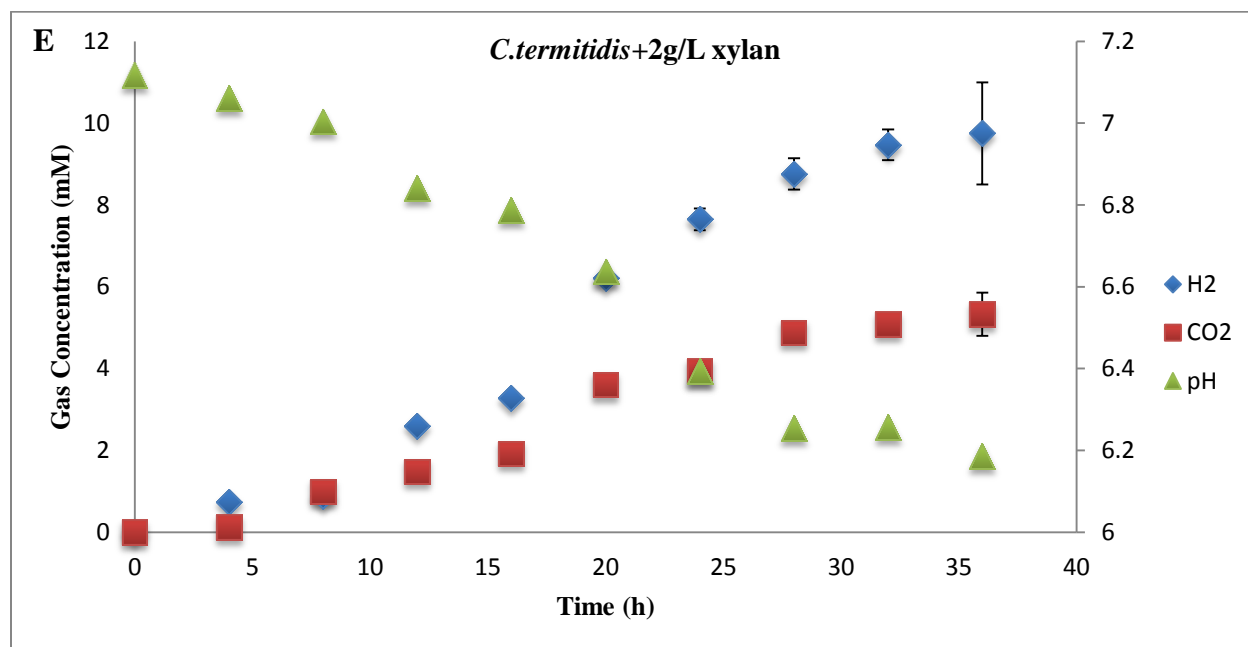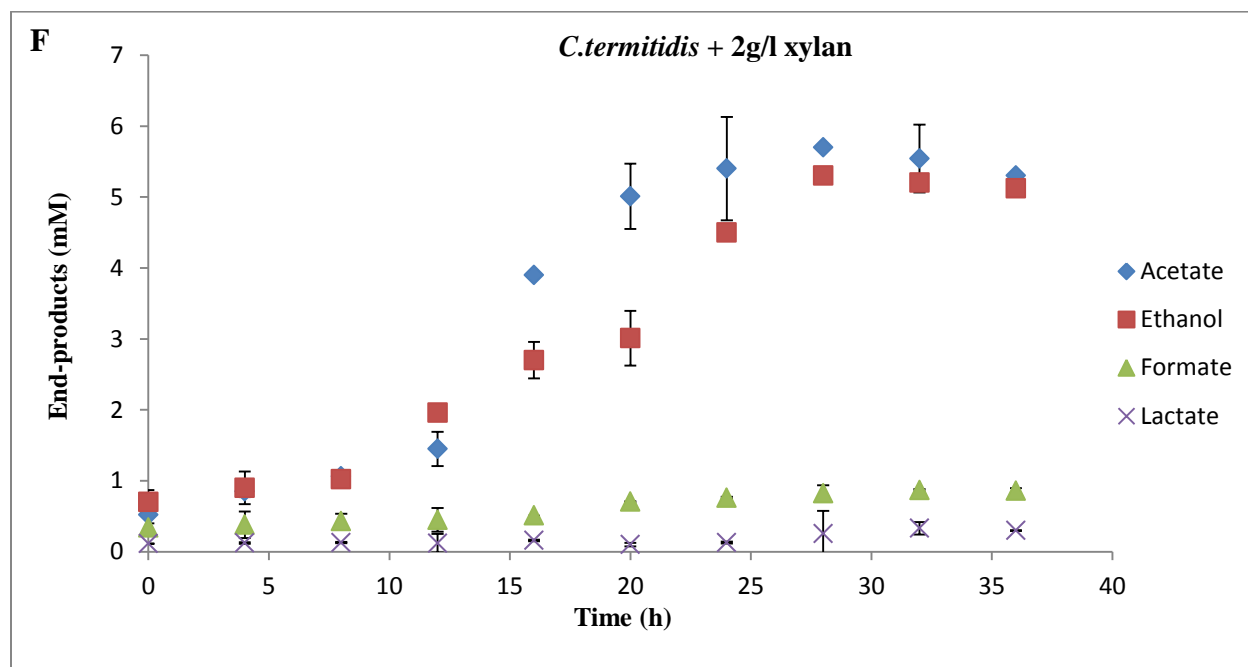

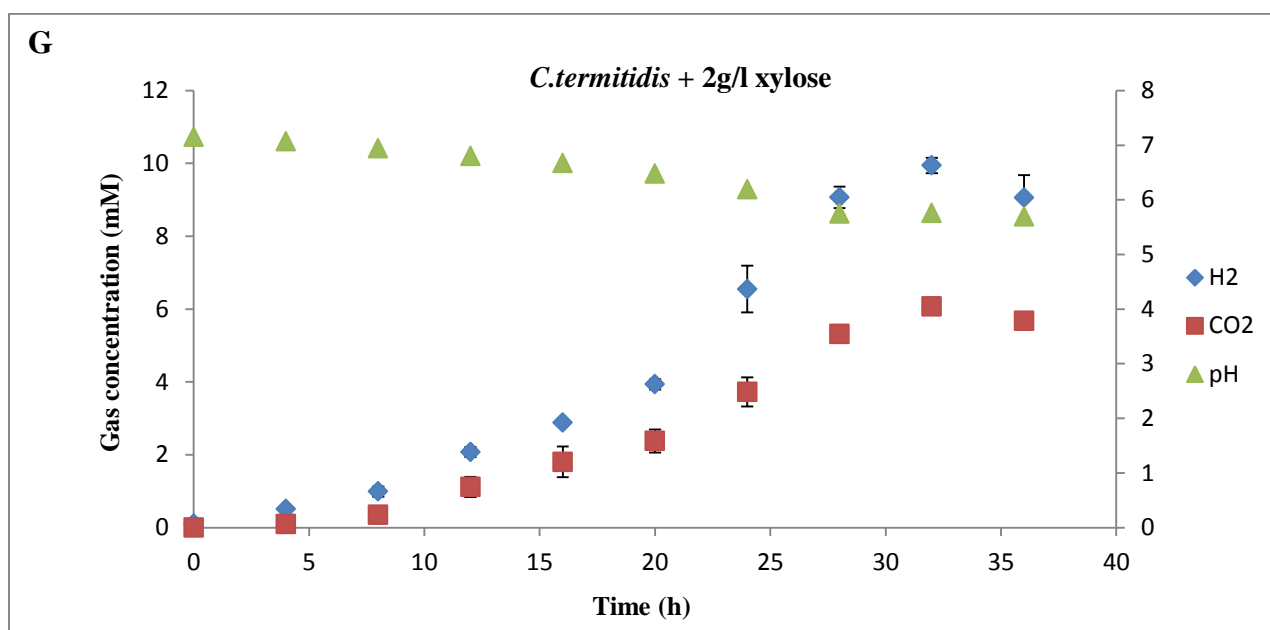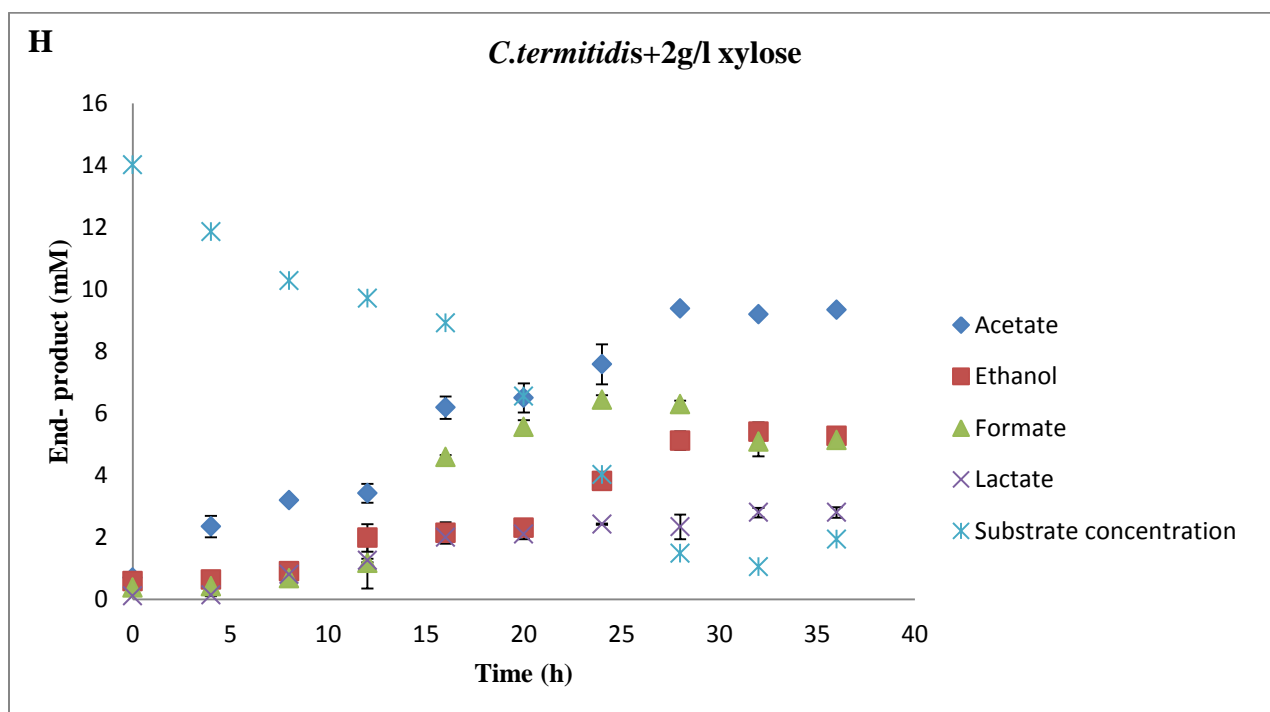

Supplement: Additional file 1: — End-product synthesis patterns in cultures of C. termitidis containing 2 g/L α-cellulose, cellobiose, xylan, and xylose. Gas production (A) and soluble end-products (B) in α-cellulose cultures; Gas production (C) and soluble end-products (D) in cellobiose cultures; Gas production (E) and soluble end-products (F) in xylan culture; Gas production (G) and soluble end-products (H) in xylose cultures Bars above and below the means represent standard deviation between replicates. (PDF 253 kb) [file 12866_2016_711_MOESM1_ESM.pdf]

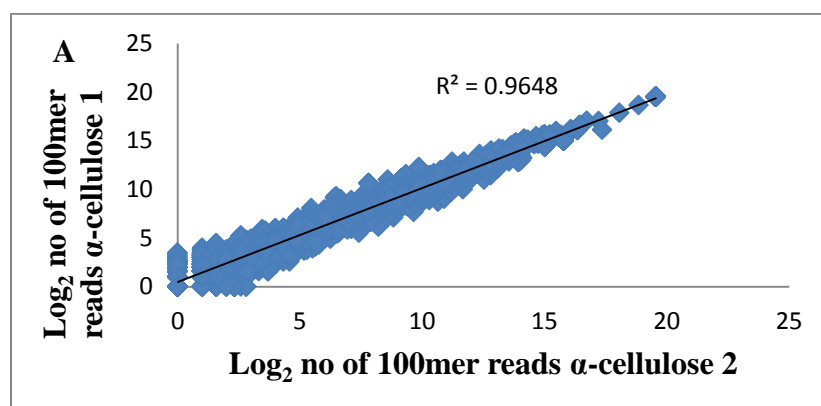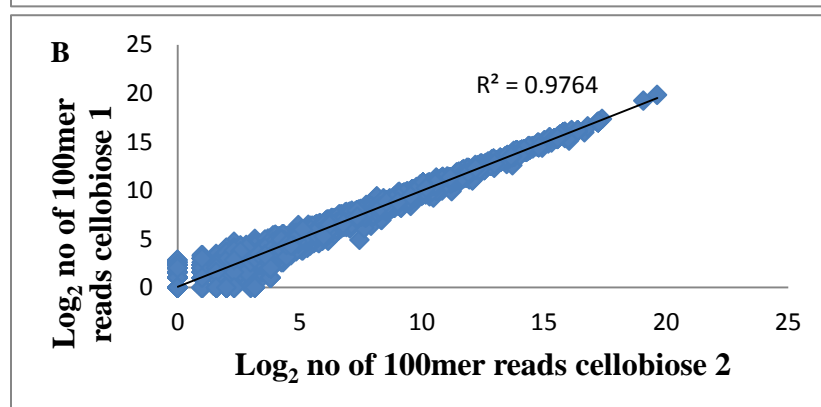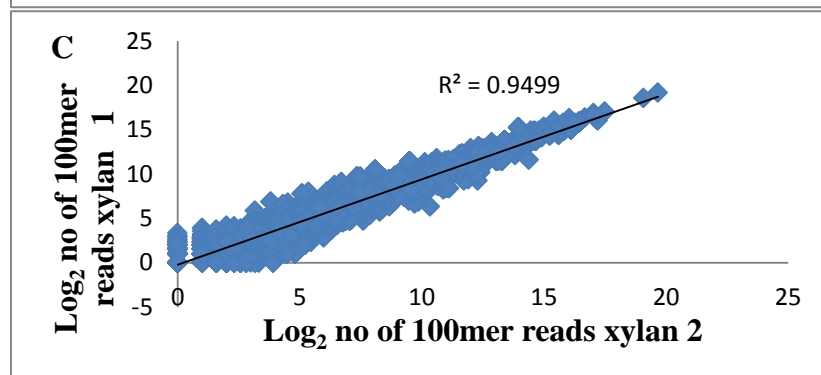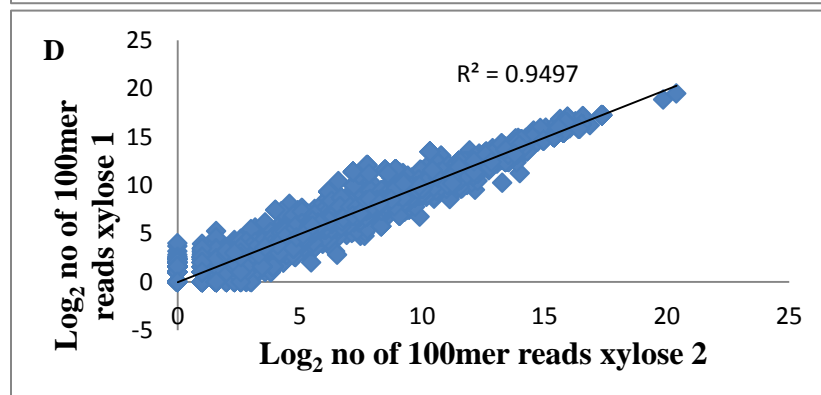

Supplement: Additional file 2: — Linear regression analysis of the sum of log2 100-mer reads per gene, between biological replicates. Values given for replicates grown on 2 g/L each α-cellulose (A); cellobiose (B); xylan (C); and xylose (D). Values observed for biological replicate 1 are plotted on the x-axis, while the corresponding values for biological replicate 2 are plotted on the y-axis. (PDF 1010 kb) [file 12866_2016_711_MOESM2_ESM.pdf]

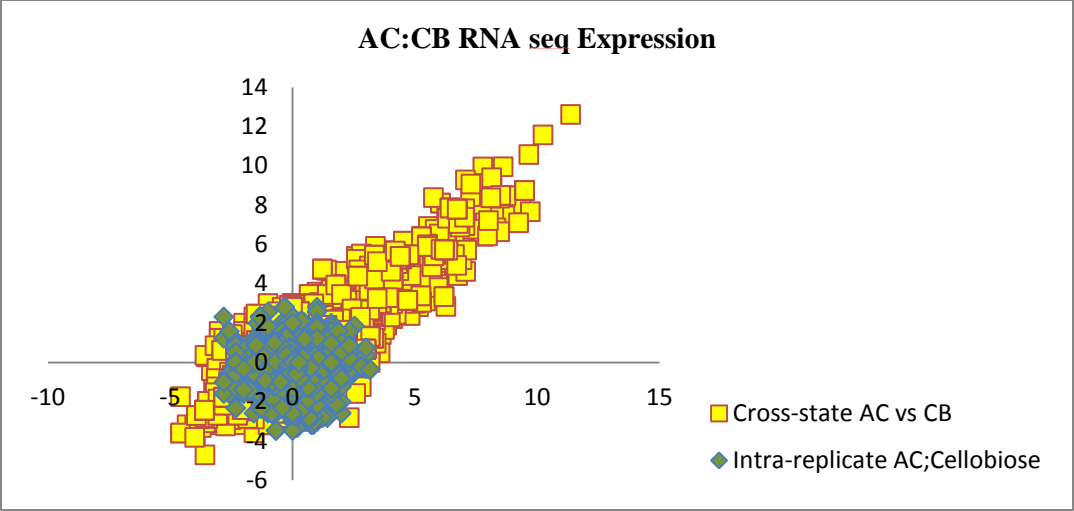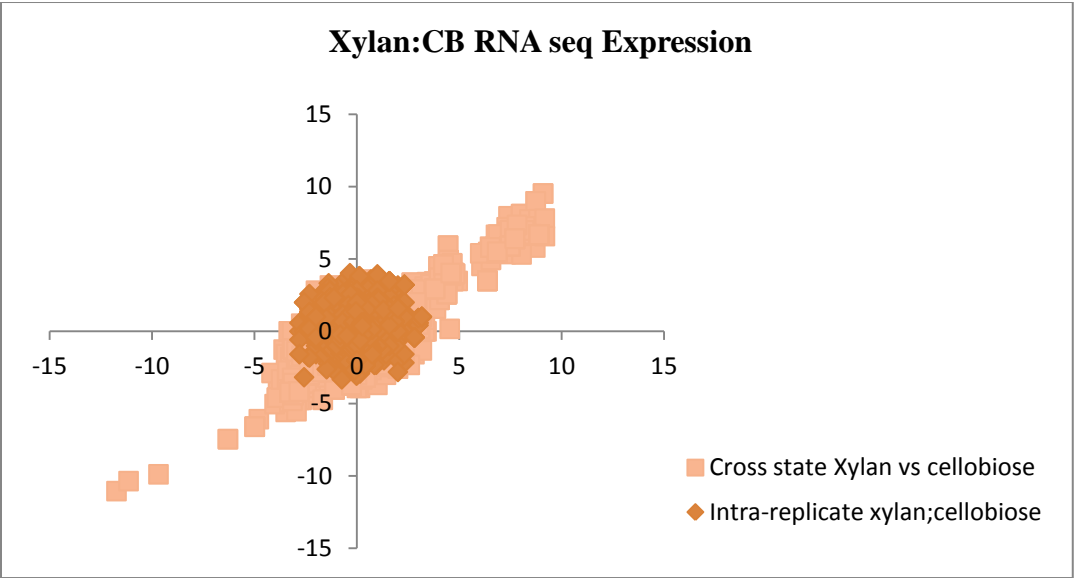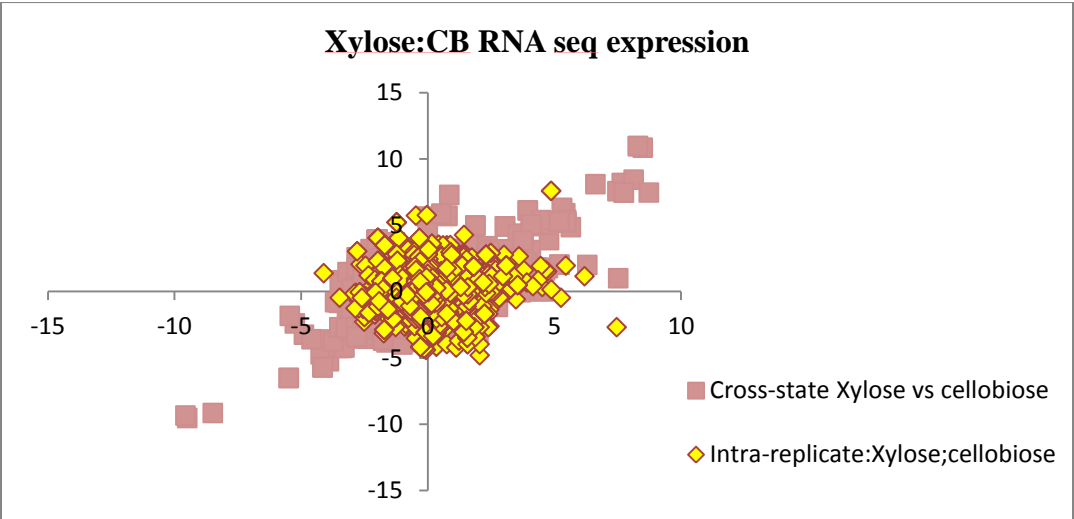

Supplement: Additional file 3: — Scatter plots showing differential RNA seq Z-scores between replicates and across state (substrates). Cross state analysis was carried out against cellobiose in all cases. AC: α-cellulose; CB: cellobiose. Values plotted along axis are from both biological replicates. (PDF 917 kb) [file 12866_2016_711_MOESM3_ESM.pdf]

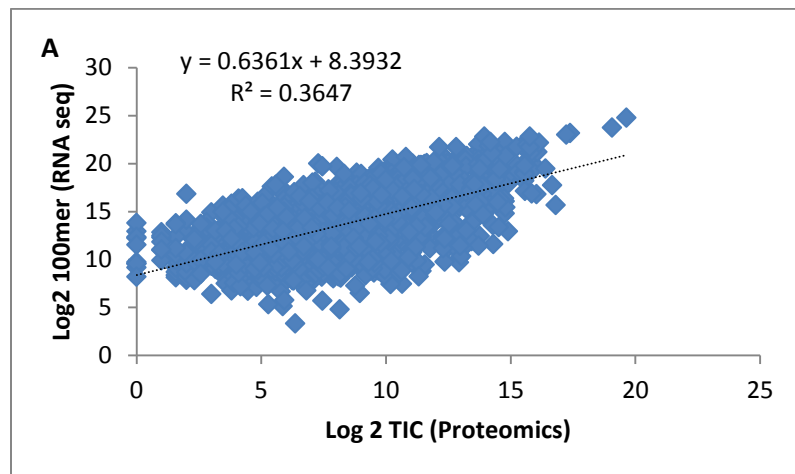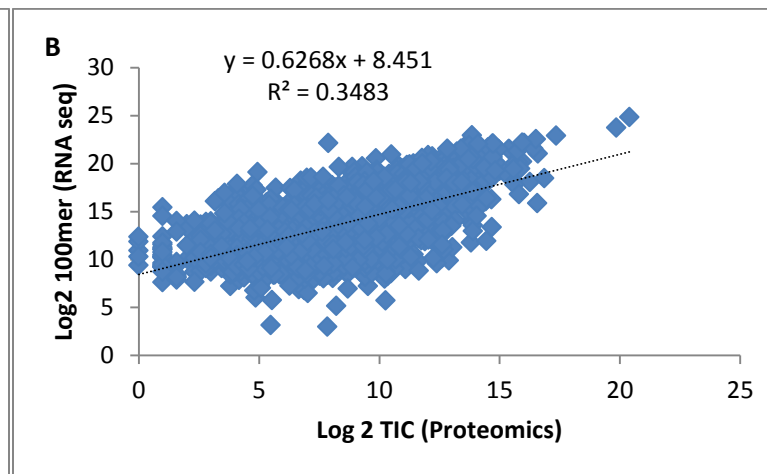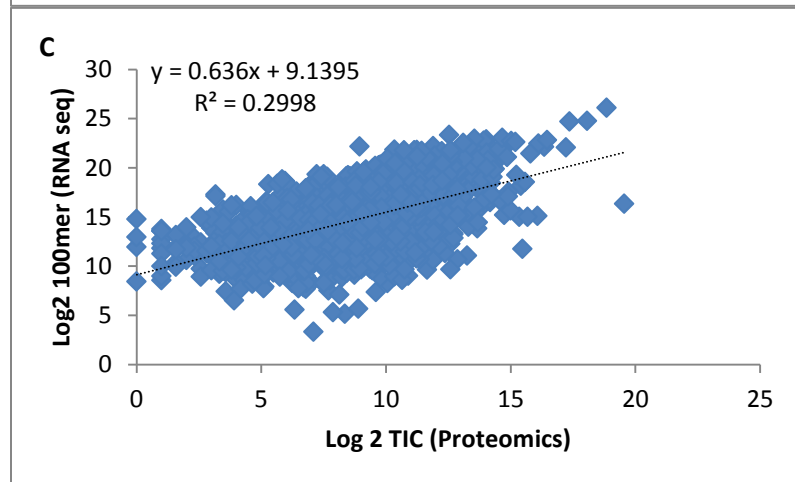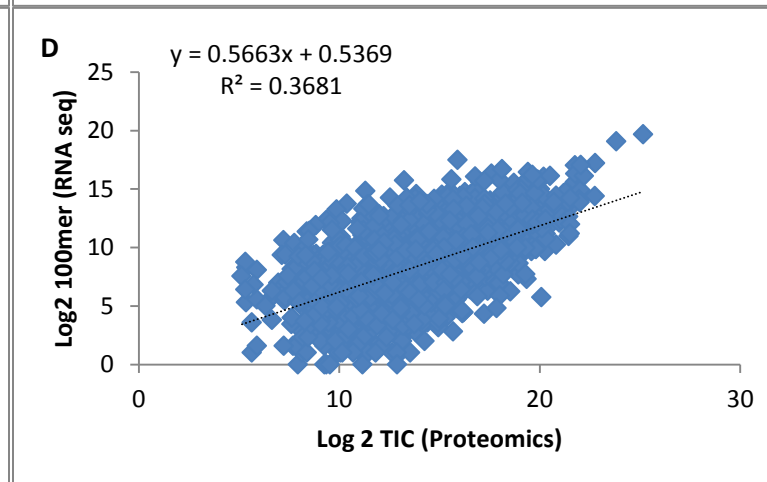

Supplement: Additional file 4: — Correlation of log2 expression values of transcriptomic and proteomic under the four experimental conditions. A: Cellobiose; B: Xylose; C: α-cellulose; D: Xylan (PDF 426 kb) [file 12866_2016_711_MOESM4_ESM.pdf]
