# Supplementary material for: Transcriptomic and proteomic analyses of core metabolism in Clostridium termitidis CT1112 during growth on α-cellulose, xylan, cellobiose and xylose
Source: BMC Microbiol. 2016 May 23;16:91. doi: 10.1186/s12866-016-0711-x (PMC4877739; doi:10.1186/s12866-016-0711-x)
Supplement: Additional file 5: — Overall biological signal to systematic noise ratio of RNAseq analyses under four experimental conditions. AC: α-cellulose; CB: cellobiose; Rep: replicate (PDF 85 kb) [file 12866_2016_711_MOESM5_ESM.pdf]

	**Comparison**	**Standard deviation (RNA seq)**
Biological signal	Xylose Rep 1 vs CB Rep 1	1.05
	Xylose Rep 2 vs CB Rep 2	1.08
	AC Rep 1 vs CB Rep 1	1.73
	AC Rep 2 vs CB Rep 2	1.59
	Xylan Rep 1 vs CB Rep 1	1.22
	Xylan Rep 2 vs CB Rep 2	1.24
System noise	CB Rep 1 vs CB Rep 2	0.58
	Xylose Rep 1 vs Xylose Rep 2	0.8
	AC Rep 1 vs AC Rep 2	0.6
	Xylan Rep 1 vs Xylan Rep 2	0.82
Signal to noise ratio for each comparing condition	Xylose vs CB Rep 1, 2	1.54
	AC vs CB Rep 1, 2	2.81
	Xylan vs CB Rep 1, 2	1.76
