# Supplementary material for: Transcriptomic and proteomic analyses of core metabolism in Clostridium termitidis CT1112 during growth on α-cellulose, xylan, cellobiose and xylose
Source: BMC Microbiol. 2016 May 23;16:91. doi: 10.1186/s12866-016-0711-x (PMC4877739; doi:10.1186/s12866-016-0711-x)
Supplement: Additional file 10: — Gas production and soluble end-product synthesis patterns of C.termitidis cultured on 1 g/L cellobiose, 1 g/L xylose, and 1 g/L cellobiose plus 1 g/L xylose. Gas production (A) in 1 g/L cellobiose cultures; Gas production (B) in 1 g/L xylose cultures; Gas production (C) in 1 g/L cellobiose plus 1 g/L xylose cultures; soluble end products (D) in 1 g/L cellobiose cultures; soluble end products (E) in 1 g/L xylose cultures; soluble end products (F) in 1 g/L cellobiose plus 1 g/L xylose culture. Bars above and below the means represent standard deviation between replicates. (PDF 150 kb) [file 12866_2016_711_MOESM10_ESM.pdf]

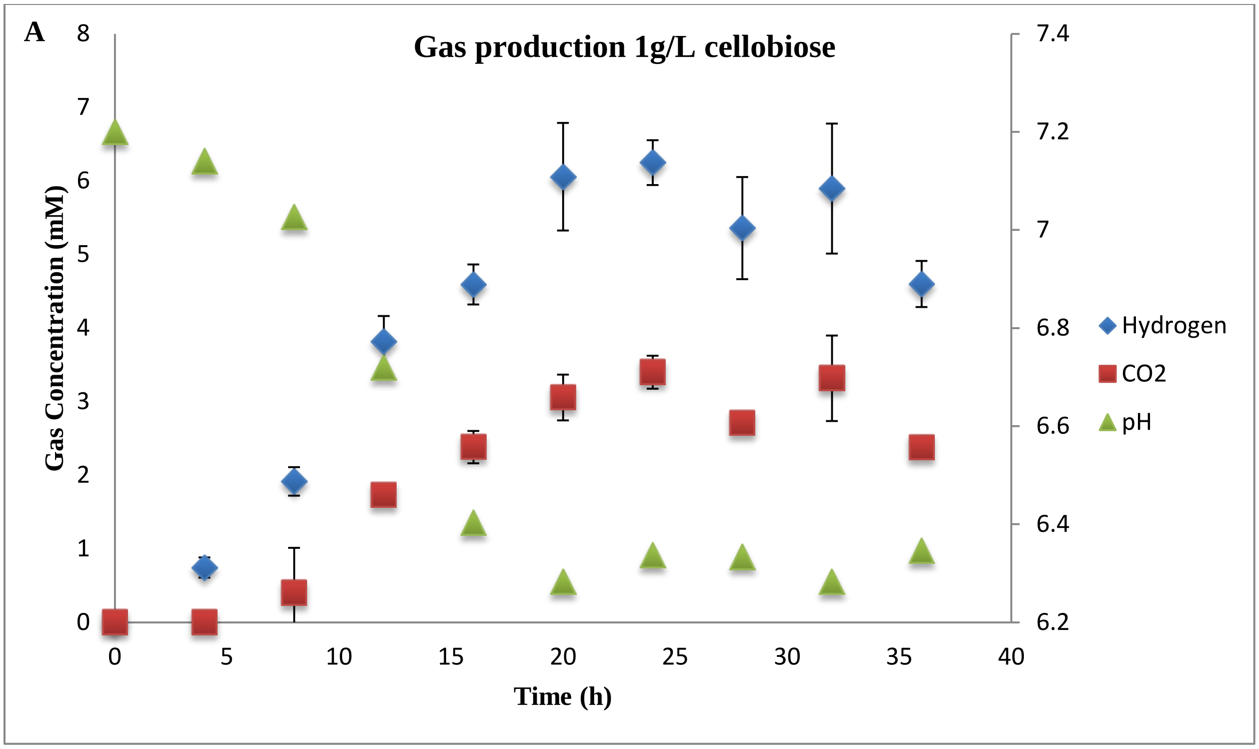
A
Gas production 1g/L cellobiose
Gas Concentration (mM)
Time (h)
Hydrogen
CO2
pH
0
1
2
3
4
5
6
7
8
6.2
6.4
6.6
6.8
7
7.2
7.4
0
5
10
15
20
25
30
35
40

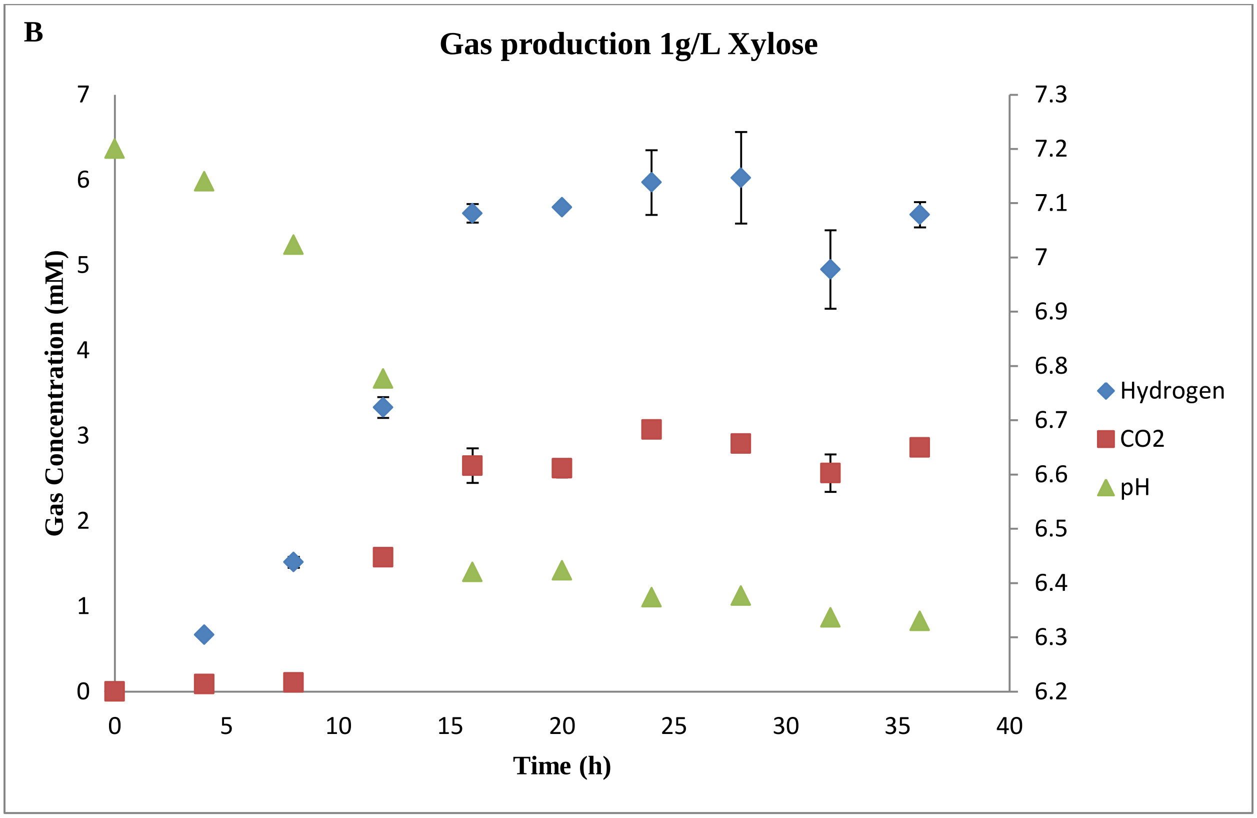
B
Gas production 1g/L Xylose
Gas Concentration (mM)
Time (h)
Hydrogen
CO2
pH
0
1
2
3
4
5
6
7
6.2
6.3
6.4
6.5
6.6
6.7
6.8
6.9
7
7.1
7.2
7.3
0
5
10
15
20
25
30
35
40

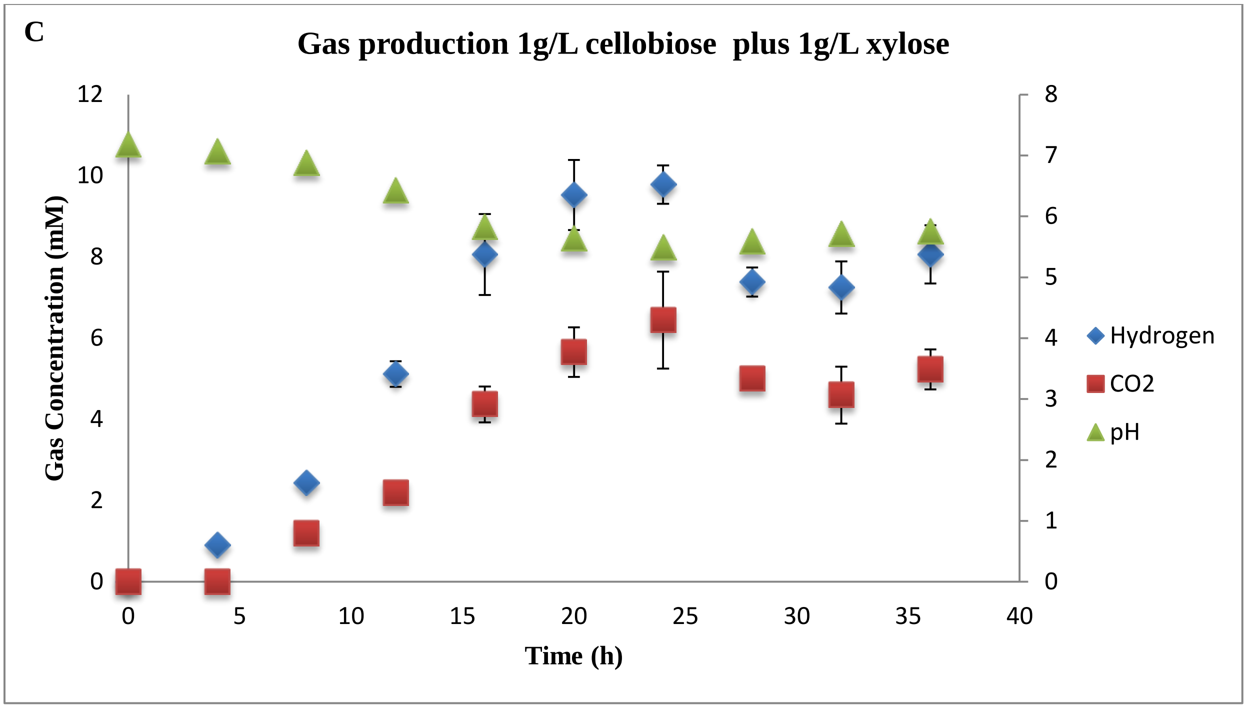
C
Gas production 1g/L cellobiose plus 1g/L xylose
Gas Concentration (mM)
Time (h)
Hydrogen
CO2
pH
0
2
4
6
8
10
12
0
5
10
15
20
25
30
35
40
0
1
2
3
4
5
6
7
8

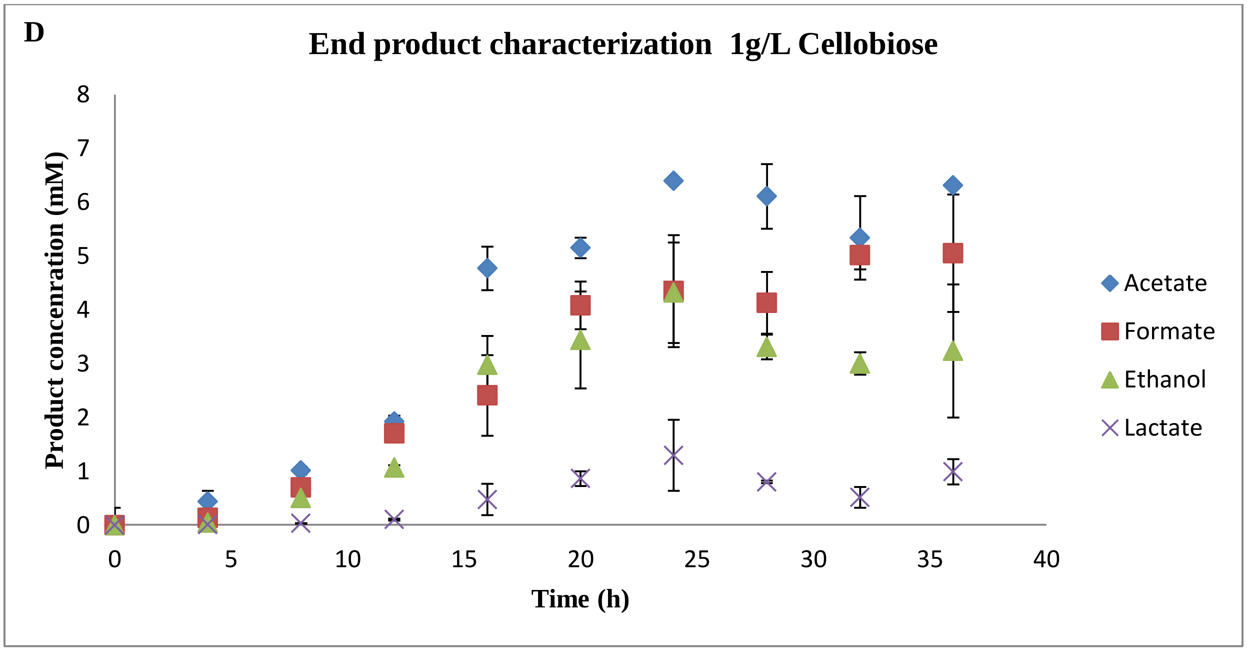
D
End product characterization 1g/L Cellobiose
Product concenration (mM)
Time (h)
Acetate
Formate
Ethanol
Lactate
0
1
2
3
4
5
6
7
8
0
5
10
15
20
25
30
35
40

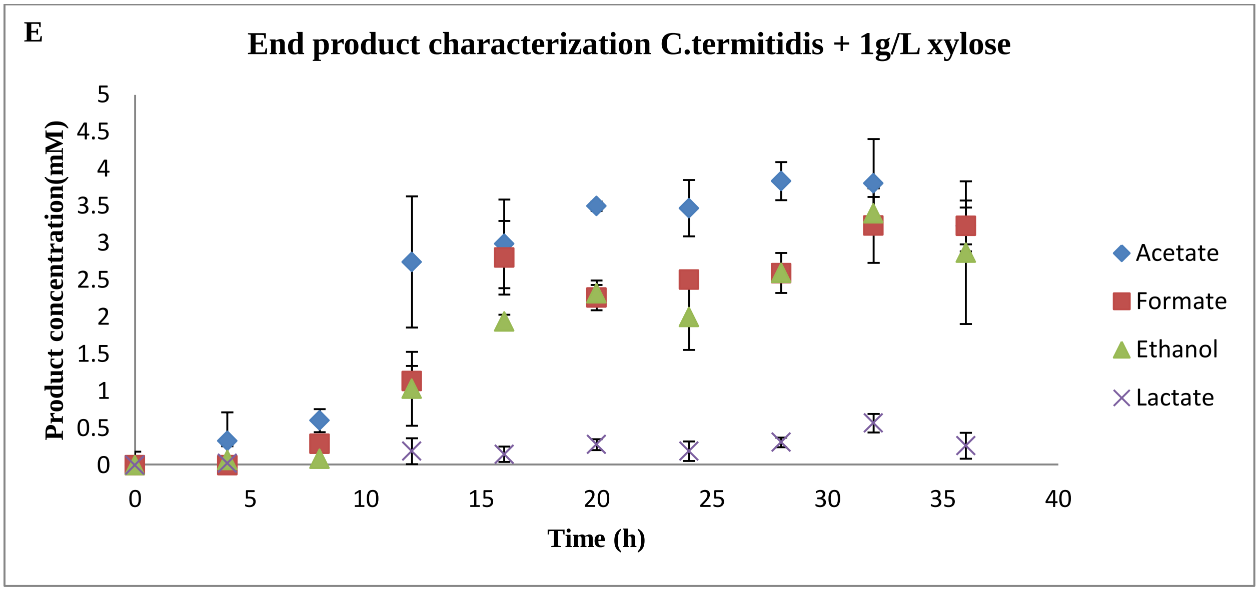
E
End product characterization C.termitidis + 1g/L xylose
Product concentration(mM)
5
4.5
4
3.5
3
2.5
2
1.5
1
0.5
0
0
5
10
15
20
25
30
35
40
Time (h)
Acetate
Formate
Ethanol
Lactate

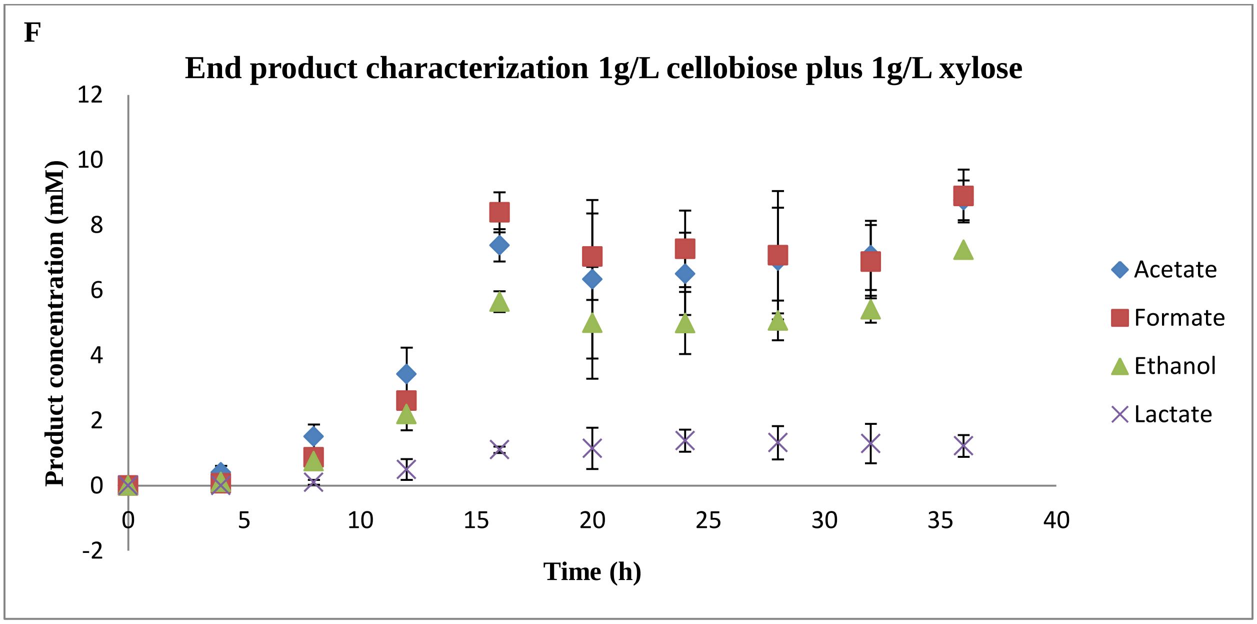
F
End product characterization 1g/L cellobiose plus 1g/L xylose
Product concentration (mM)
12
10
8
6
4
2
0
-2
0
5
10
15
20
25
30
35
40
Time (h)
Acetate
Formate
Ethanol
Lactate
